# MicroRNA 26a (miR-26a)/KLF4 and CREB-C/EBPβ regulate innate immune signaling, the polarization of macrophages and the trafficking of *Mycobacterium tuberculosis* to lysosomes during infection

**DOI:** 10.1371/journal.ppat.1006410

**Published:** 2017-05-30

**Authors:** Sanjaya Kumar Sahu, Manish Kumar, Sohini Chakraborty, Srijon Kaushik Banerjee, Ranjeet Kumar, Pushpa Gupta, Kuladip Jana, Umesh D. Gupta, Zhumur Ghosh, Manikuntala Kundu, Joyoti Basu

**Affiliations:** 1 Department of Chemistry, Bose Institute, Kolkata, India; 2 Bioinformatics Centre, Bose Institute, Kolkata, India; 3 National JALMA Institute for Leprosy and Other Mycobacterial Diseases, Tajganj, Agra, India; 4 Division of Molecular Medicine, Bose Institute, Kolkata, India; New Jersey Medical School, UNITED STATES

## Abstract

For efficient clearance of *Mycobacterium tuberculosis* (Mtb), macrophages tilt towards M1 polarization leading to the activation of transcription factors associated with the production of antibacterial effector molecules such as nitric oxide (NO) and proinflammatory cytokines such as interleukin 1 β (IL-1β) and tumor necrosis factor α (TNF-α). At the same time, resolution of inflammation is associated with M2 polarization with increased production of arginase and cytokines such as IL-10. The transcriptional and post-transcriptional mechanisms that govern the balance between M1 and M2 polarization, and bacteria-containing processes such as autophagy and trafficking of Mtb to lysosomes, are incompletely understood. Here we report for the first time, that the transcription factor KLF4 is targeted by microRNA-26a (miR-26a). During Mtb infection, downregulation of miR-26a (observed both *ex vivo* and *in vivo*) facilitates upregulation of KLF4 which in turn favors increased arginase and decreased iNOS activity. We further demonstrate that KLF4 prevents trafficking of Mtb to lysosomes. The CREB-C/EBPβ signaling axis also favors M2 polarization. Downregulation of miR-26a and upregulation of C*/ebpbeta* were observed both in infected macrophages as well as in infected mice. Knockdown of *C/ebpbeta* repressed the expression of selected M2 markers such as *Il10* and *Irf4* in infected macrophages. The importance of these pathways is substantiated by observations that expression of miR-26a mimic or knockdown of *Klf4* or *Creb* or *C/ebpbeta*, attenuated the survival of Mtb in macrophages. Taken together, our results attribute crucial roles for the miR-26a/KLF4 and CREB-C/EBPβsignaling pathways in regulating the survival of Mtb in macrophages. These studies expand our understanding of how Mtb hijacks host signaling pathways to survive in macrophages, and open up new exploratory avenues for host-targeted interventions.

## Author summary

The ability of *Mycobacterium tuberculosis* (Mtb) to establish infection depends on its ability to evade the early innate immune defense mechanisms, exploiting the transcriptional and post-transcriptional regulatory mechanisms of host macrophages. We have used the macrophage infection model to understand Mtb-triggered innate immune responses. We have coupled global gene expression analysis and microRNA profiling previously carried out in our laboratory to understand transcriptional and post-transcriptional networks associated with infection of macrophages by Mtb. We demonstrate how Mtb exploits macrophage signaling pathways to orchestrate a response which dictates a bias towards M2 polarization, decreased expression of antibacterial effectors such as NO and decreased trafficking of Mtb to lysosomes. miR-26a targets KLF4. KLF4 tilts the macrophage response towards production of arginase and inhibition of autophagy. Further, CREB-dependent synthesis of C/EBPβ also favors the production of arginase as well as markers of M2 polarization such as IL-10. The miR-26a/KLF4 and CREB-C/EBPβ signaling cascades converge upon pathways that are of benefit to bacterial survival in its intracellular niche. These crucial findings should in the long run, add value to the efforts towards developing host-targeted therapeutic strategies.

## Introduction

Tuberculosis (TB) caused by the pathogen *Mycobacterium tuberculosis* (Mtb) is a global health problem. According to the World Health Organization’s global tuberculosis report of 2015, 9.6 million people fell ill with TB in 2014[1]. In order to circumvent the problems of drug resistance, host directed therapies are increasingly being recognized as alternate strategies for combating TB. Such approaches require understanding of the interplay between pathogen and host. Macrophages are central to the control of TB. However, the bacterium resides within this bactericidal niche by co-opting host pathways and processes for its own benefit. It is therefore of utmost importance to understand the dynamic regulation of the macrophage response to Mtb infection and to identify key molecules which dictate the balance in the crosstalk between the host and the pathogen.

A simplified view of macrophage polarization suggests that classical activation triggers differentiation of macrophages into the M1 phenotype characterized by the production of inflammatory cytokines such as tumor necrosis factor α (TNFα) and interleukin (IL)-6, as well as reactive oxygen and nitrogen species. On the other hand, alternate activation generates M2 macrophages which produce anti-inflammatory cytokines such as IL-10 and show increased expression of arginase 1 (Arg1) [2]. Arginine metabolism is central to the M1/M2 balance [3]. The balance between the enzymes inducible nitric oxide synthase (iNOS) and Arg1 is tilted in favor of the former in M1, and in favor of the latter in M2 macrophages. Loss of Arg1 is associated with low bacterial burdens in the lungs of mice [4,5]. Lung granulomas in mice lacking Arg1 occupy a smaller fraction of the total lung area compared to the wild type lungs. In the light of these observations, it is particularly relevant to understand the repertoire of transcription factors that regulate the iNOS/arginase balance and the balance between pro- and anti-inflammatory cytokines.

The family of Krüppel-like factors (KLFs) have 17 members with highly conserved C-terminal DNA binding domains consisting of three C2H2 zinc fingers that bind to GC-box or CACCC-box motifs [6]. The non DNA-binding regions are divergent and mediate interactions with co-regulators. KLF4 binds DNA in heterochromatin converting chromatin to an open form and facililitates binding of other transcription factors. Several of the KLFs play important roles in host-pathogen interactions and macrophage polarization [7–12]. KLF4 inhibits M1 and activates M2 polarization [10, 11].

C/EBPβ is one among several transcription factors in addition to KLF4, which activates M2 polarization. It has two isoforms. The full length form, LAP usually activates gene expression, whereas the shorter isoform LIP, lacking the transactivation domain, usually performs a repressive function[13,14]. C/EBPβ has a context-specific role in inflammation and M1/M2 polarization [15, 16]. BCG-induced arginase 1 expression in macrophages is dependent on C/EBPβ [17]. In response to IL-4, STAT6 and C/EBPβ assemble at an enhancer element upstream of the basal arginase 1 promoter [18,19]. C/EBPβ is therefore likely to be a pivotal transcription factor in dictating the M1/M2 balance during Mtb infection. The transcription factor CREB is an activator of C/EBPβ expression [20].

MicroRNAs (miRNAs) are small RNAs of 22 nucleotides which regulate gene expression to modulate biological processes including immune cell development and immune-related functions [21–24]. Base complementarity between an miRNA and its target usually involves a sequence located in the 3’-untranslated region (3’-UTR) of the target. Identical base pairing generally results in mRNA cleavage, whereas imperfect base pairing results in translational repression of the mRNA target [25–28]. MicroRNAs have emerged as key regulators of the response of macrophages to Mtb infection; controlling inflammation [29, 30] recruitment of neutrophils to the lung [31] and autophagy [32, 33], among other processes.

In this paper, we have analyzed the transcriptome of Mtb-infected macrophages, listed the transcription factors (TFs) that are differentially regulated during infection, and narrowed down on a set of TFs that are reportedly involved in dictating the balance of M1/M2 polarization. Specifically, the TFs KLF4 and C/EBPβ were transcriptionally downregulated and upregulated respectively during Mtb infection. However, expression of the KLF4 protein was enhanced during infection, indicating uncoupling of transcriptional and translational regulation. We report that an miRNA-driven mechanism is linked to this uncoupling. We validate that KLF4 is a target of miR-26a-5p (hereafter referred to as miR-26a) in RAW264.7 and primary mouse and human macrophages. We have linked the downregulation of miR-26a during infection with the translational upregulation of KLF4. The downregulation of miR-26a was further confirmed in the lungs, spleen and lymph nodes of Mtb-infected mice. In addition to KLF4, upregulation of C/EBPβ and CREB also favors M1 to M2 polarisation with an increased production of M2 markers and decreased production of M1 markers in Mtb infected macrophages. Importantly, KLF4 upregulates Mcl-1 expression thereby repressing autophagy during Mtb infection as well as the trafficking of Mtb to lysosmes. In summary, we establish how Mtb infection triggers transcriptional and post-transcriptional signaling pathways in macrophages that converge to favor M2 polarization and repress autophagy, both associated with increased survival of the pathogen.

## Results

### The transcription factors KLF4 and C/EBPβ are differentially regulated in Mtb-infected macrophages

**Fig 1:**
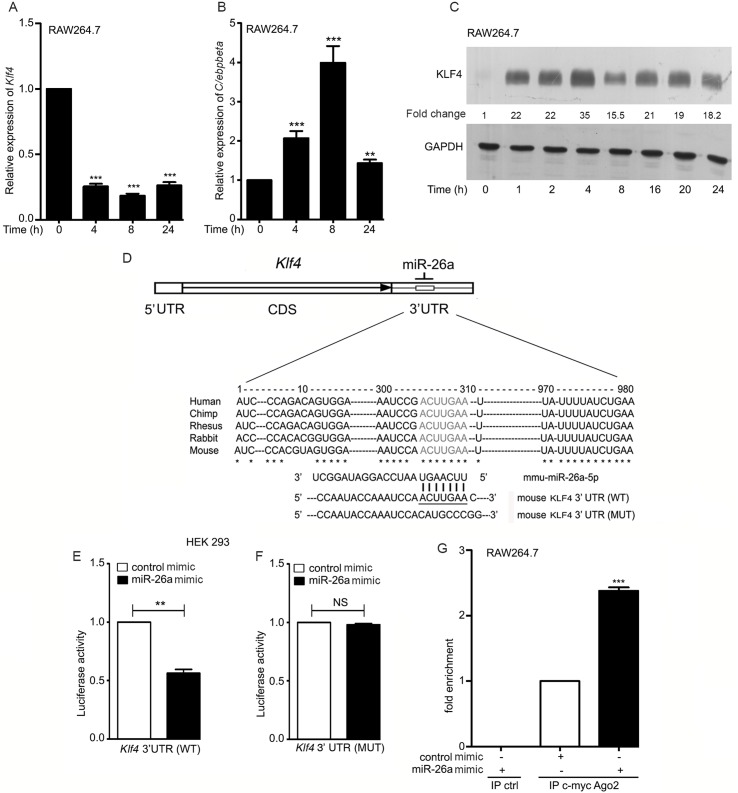
KLF4 and C/EBPβ are differentially regulated in *Mycobacterium tuberculosis* (Mtb)-infected macrophages and KLF4 is targeted by miR-26a. (A,B) RAW264.7 cells were infected with Mtb at an MOI of 10 for 24 h, RNA was isolated and the relative expression of *Klf4* (A) and *C/ebpbeta* (B) were analyzed with respect to uninfected cells (normalized to 1). (C) Time-dependent expression of KLF4 was analyzed by Western blotting in Mtb-infected RAW264.7. Intensities of bands were measured by densitometric scanning. Each data point is represented as ratio of intensities of KLF4 and GAPDH (indicated between the two blots). (D)Schematic representation of *Klf4* mRNA showing the relative positions of coding sequence and 3′-UTR regions (not to scale). (E,F) HEK293 cells were co-transfected with plasmids expressing *Klf4* 3′UTR (wild type, WT) (E) or mutant, MUT) (F) and β-galactosidase, along with miR-26a mimic (or control mimic). After 24 h of transfection, luciferase assays were performed. Readings were normalized to β-galactosidase activities. (G) Association of miR-26a with *Klf4*. RAW 264.7 cells were cotransfected with c-*myc*-Ago2 in the presence or absence of miR-26a mimic for 24 h followed by lysis of cells and immunoprecipitation with either anti-c-*myc*-agarose or protein A/G-agarose (ctrl). qRT-PCR was performed to confirm the presence of *Klf4* in the immunoprecipitates of cells transfected with miR-26a. Results in panels A,B, E-G represent means ± SEM (n = 3). The blot in (C) is representative of three experiments. ** *p<*0.01; *** *p<*0.001.

A heat map of transcription factors (TFs) that are differentially regulated during Mtb infection, was generated from the analysis of global gene expression profiles during Mtb infection of RAW264.7 (S1 Fig). Macrophage polarization is transcriptionally regulated by discrete sets of TFs [10, 34]. From the heat map, we focused selectively on two transcription factors which regulate M1/M2 polarization in macrophages, KLF4 and C/EBPβ. *Klf4* was downregulated and *C/ebpbeta* was upregulated in macrophages, 24 h post-infection. We validated the gene expression data by qRT-PCR of *Klf4* and *C/ebpbeta* expression in infected macrophages (Fig 1A and 1B). We then checked the expression of KLF4 at the level of protein. Intriguingly, the expression of the KLF4 protein was upregulated during infection (Fig 1C). Several post-transcriptional regulatory mechanisms could potentially be associated with the inverse relationship between KLF4 mRNA and protein levels. Since increase in KLF4 expression during infection could not be linked to transcriptional regulation, we investigated a possible miRNA-dependent mechanism of translational upregulation of KLF4. We searched for an inverse relationship between KLF4 and miRNA expression during infection. Taqman Low Density Array (TLDA)-based miRNA profiling provided a list of miRNAs that are downregulated in Mtb-infected RAW264.7. We narrowed down to miRNAs with putative binding sites in the *Klf4* 3’-UTR. Using TARGETSCAN and Pic Tar, a list of predicted *Klf4*-targeting miRNAs was compiled (S1 Table) with the inclusion criteria that (a) the miRNA was predicted to target *Klf4* using both the algorithms; (b)it was perfectly conserved in humans and in mice and (c) it was downregulated both at 4 and 24 h of infection. miR-26a was considerably downregulated both at 4 and 24 h post-infection. mmu-miR-200b-3p, miR-200c-3p and miR-26b-5p were downregulated to a lesser extent than miR-26a at 4 h post-infection. The extent of downregulation of miR-128-3p was much less than that of miR-26a at 24 h post-infection. Considering that KLF4 levels remained well above basal levels even at 24 h post-infection (Fig 1C), we narrowed down on miR-26a. The seed sequence for miR-26a binding was conserved in the *Klf4*3’UTR across several species (Fig 1D). HEK293 cells were transfected with a luciferase-based reporter plasmid harboring a constitutive luciferase gene fused to the *Klf4* 3’UTR. Cotransfection of a miR-26a mimic, inhibited the luciferase activity (Fig 1E). Mutating the miR-26a seed sequence in the *Klf4* 3’UTR, relieved the fused luciferase gene from the repressive effects of miR-26a (Fig 1F). These results suggested that miR-26a targets the *Klf4* 3’UTR. In order to test whether *Klf4* is a bona fide target of miR-26a we tested whether *Klf4* associates with the RNA-induced silencing complex (RISC) upon overexpression of miR-26a. Based on the principle that the miRNA acts as a template for binding complementary sequences of mRNA and activates Argonaute (Ago), we transfected c-Myc-tagged Ago2-expressing construct and either a control or an miR-26a mimic. Immunoprecipitation with Myc-agarose followed by qRT-PCR for *Klf4*, confirmed enrichment of *Klf4* in the immunoprecipitates (Fig 1G). Taken together, these data supported the view that *Klf4* is a bona fide miR-26a target.

### The *Klf4*-targeting miRNA, miR-26a is differentially regulated during Mtb infection

**Fig 2:**
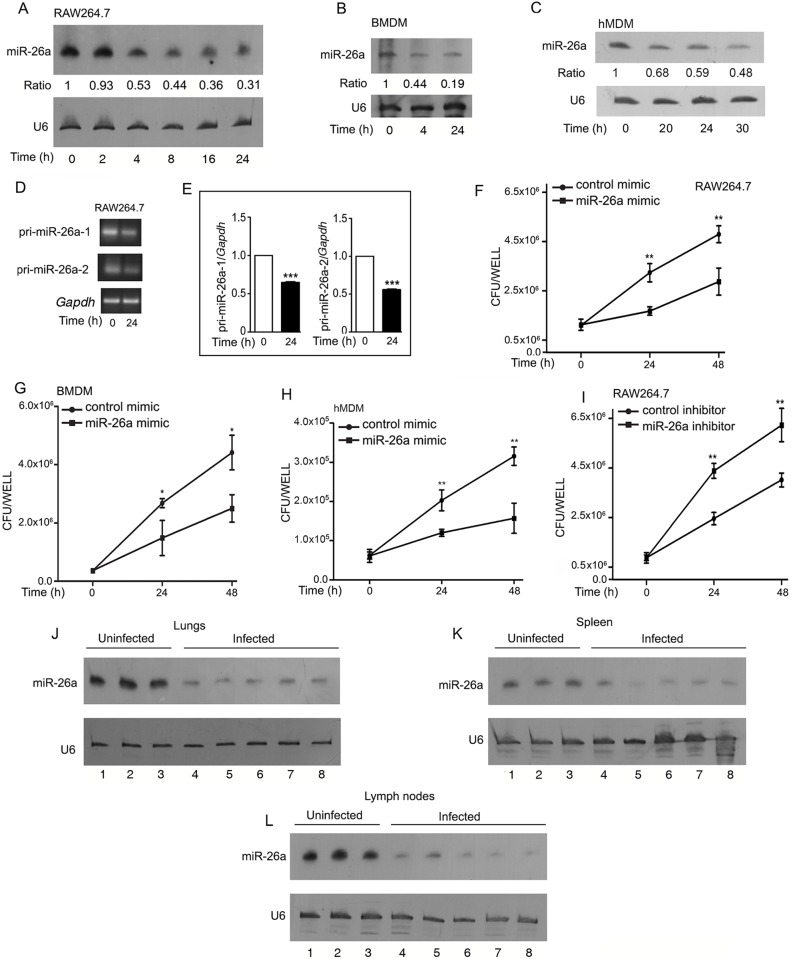
miR-26a is downregulated during Mtb infection and regulates the survival of Mtb in macrophages. (A-E) RAW264.7 cells (A, D, E) or BMDMs (B) or hMDMs (C) were infected with Mtb for different periods of time as indicated in the figure. RNA was isolated and expression of miR-26a (A-C) or pri-miR-26a-1 or pri-miR-26a-2 (D,E) was assessed by Northern blotting (A-C) using U6 for normalization; or by semi-quantitative RT-PCR (D, E) using *Gapdh* for normalization. (F-I) The effect of miR-26a on bacterial survival was analyzed by transfecting RAW264.7 (F,I) or BMDMs (G) or hMDMs (H) with control mimic or miR-26a mimic (F-H), or with control inhibitor or miR-26a inhibitor (I) prior to infection with Mtb. CFUs were determined at different time periods post-infection as indicated. Results represent the means ± SEM (n = 3); *p< 0.05; ** *p<*0.01; *** *p<*0.001. (J-L) Expression of miR-26a was analysed by Northern blotting in lungs (J), spleen (K) or lymph nodes (L). The numbers on the horizontal axes of panels J-L represent individual mice (uninfected: 1–3; infected: 4–8). Intensities of bands were measured by densitometric scanning. The data is shown in S2A–S2C Fig.

Before progressing further, we validated the downregulation of miR-26a in infected macrophages by Northern blotting. miR-26a levels progressively decreased over a period of time in both infected RAW264.7, murine bone marrow-derived macrophages (BMDMs) and human monocyte-derived macrophages (hMDMs)(Fig 2A–2C). miRNAs are synthesized from a long primary transcript designated pri-miRNA. We observed that pri-miR-26a also decreased in macrophages 24 h after infection (Fig 2D and 2E). In order to gain an understanding whether the downregulation of miR-26a is of relevance to infection, we scored for bacterial CFUs in RAW264.7 or in BMDMs or in hMDMs infected with Mtb either in the presence or absence of miR-26a mimic or inhibitor. Survival of Mtb was compromised in the presence of miR-26a mimic both in murine (Fig 2F and 2G) or in human (Fig 2H) macrophages. On the other hand, it was augmented in the presence of a miR-26a inhibitor in RAW264.7 (Fig 2I). Neither the miR-26a mimic nor the miR-26a inhibitor altered cell viability during the time period studied (S2A–S2D Fig). These results suggested that miR-26a downregulation likely helps in the establishment of infection. Interestingly, miR-26a levels were lower in lungs, spleen and lymph nodes of infected animals 12 weeks after infection, compared to uninfected mice (Fig 2J–2L(Northern blots), and S2E–S2G Fig (quantification of Northern blots by densitometric scanning). The differential expression of miR-26a, suggested that this could be of likely relevance to infection.

### miR-26a regulates KLF4 during infection

**Fig 3:**
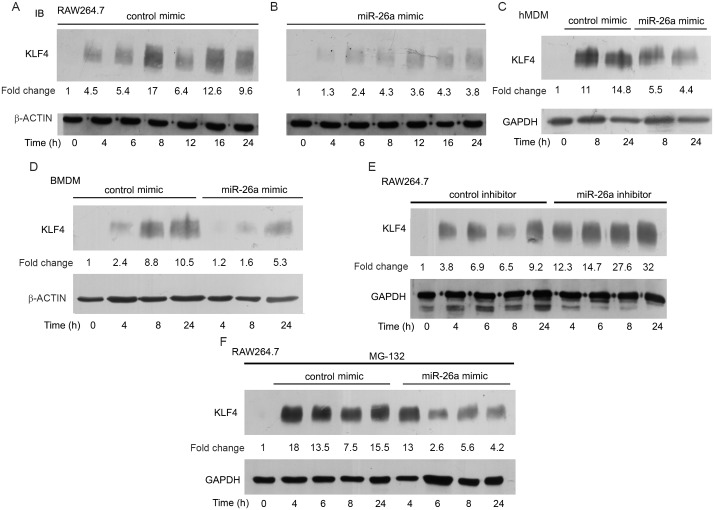
miR-26a regulates the expression of KLF4 in Mtb-infected macrophages. (A-F) RAW264.7 (A, B, E, F) or hMDMs (C), or BMDMs (D) were transfected with control mimic or with miR-26a mimic (A-D, F); or with control inhibitor or miR-26a inhibitor (E) prior to infection. (F) RAW264.7 cells were transfected with control mimic or with miR-26a mimic, then treated with MG132; prior to infection with Mtb. Time-dependent expression of KLF4 was analyzed by Western blotting. The data shown in panels A, B, D-F are representative of three independent experiments; the data shown in panel C is representative of two independent experiments. Intensities of bands were measured by densitometric scanning. The fold change was calculated with respect to uninfected cells.

In order to test whether miR-26a regulates KLF4 expression, we analyzed the levels of KLF4 protein in infected macrophages, either in the presence of a control mimic or in the presence of a miR-26a mimic. KLF4 levels increased over time in cells transfected with control mimic, but this increase was repressed in the presence of the miR-26a mimic in RAW264.7 cells, hMDMs and BMDMs (Fig 3A–3D). miR-26a was downregulated in THP-1 macrophages, and KLF4 was repressed in the presence of a miR-26a mimic in these cells also (S3 Fig). At the same time, KLF4 levels were augmented in cells infected in the presence of a miR-26a inhibitor, compared to cells infected in the presence of a control inhibitor (Fig 3E). These results clearly suggested that miR-26a downregulation during infection is one of the likely reasons for the increase in KLF4 protein levels over time. KLF4 levels are also regulated by proteasomal degradation of the protein [35]. In order to rule out the possibility that miR-26a exerts its effects via the proteasomal machinery, cells were pretreated with the proteasomal inhibitor MG132 prior to incubation with a miR-26a mimic (or control mimic). The repression in KLF4 levels associated with transfection of a miR-26a mimic prior to infection, occurred even in the presence of MG132 (Fig 3F) supporting the notion that miR-26a mediates its effects in a proteasome-independent manner.

### KLF4 and miR-26a regulate the balance between arginase and inducible nitric oxide synthase (iNOS) expression in Mtb-infected macrophages, and bacterial survival

**Fig 4:**
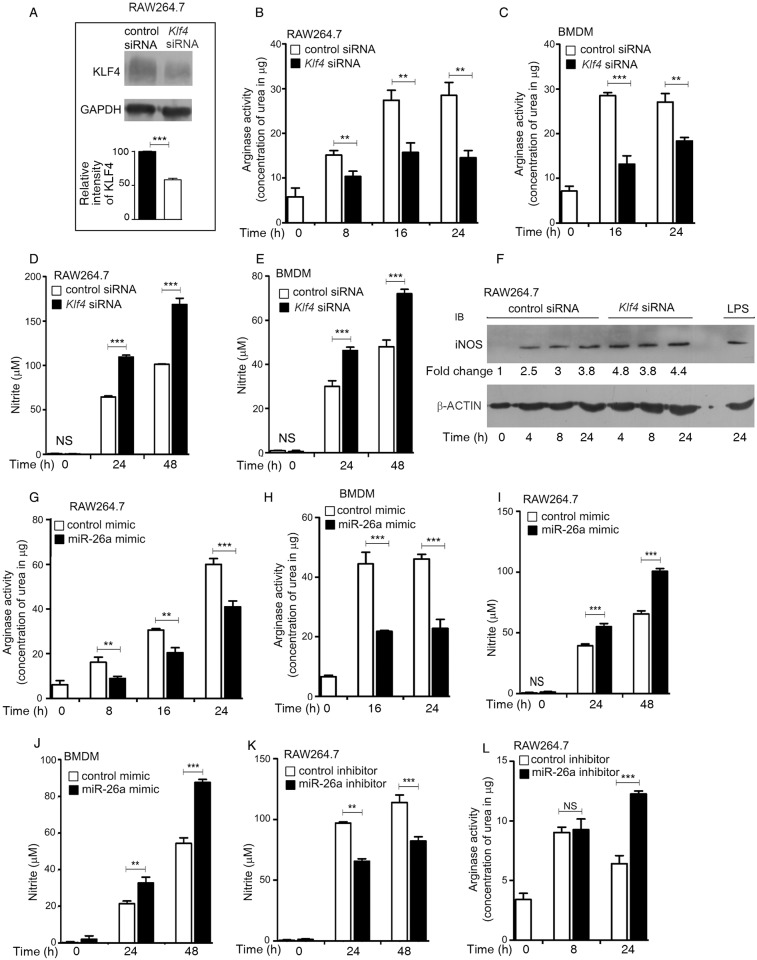
KLF4 and miR-26a regulate arginase and iNOS activities in Mtb-infected macrophages. (A-F) RAW264.7 cells (A,B,D) or BMDMs (C,E) were transfected with either control siRNA or *Klf4* siRNA for 48 h prior to infection with Mtb for different periods of time, followed by measurement of arginase activity (B,C), nitrite production (D,E) or Western blotting of lysates for KLF4(A) or iNOS (F). For B-E, results are means ± SEM, n = 3. For A and F, blots are representative of results obtained in three separate experiments. (G-L) RAW264.7 cells (G,I) or BMDMs (H,J) were transfected with control mimic or miR-26a mimic; or RAW264.7 cells were transfected with control inhibitor or miR-26a inhibitor (K,L) prior to infection with Mtb. Arginase activity (G,H,L) or nitrite production (I-K) was measured at different periods of time after infection. Results are means ± SEM, n = 3. ** *p<*0.01; *** *p<*0.001. For (F), intensities of bands were measured by densitometric scanning. The fold change in iNOS was calculated with respect to uninfected cells.

**Fig 5:**
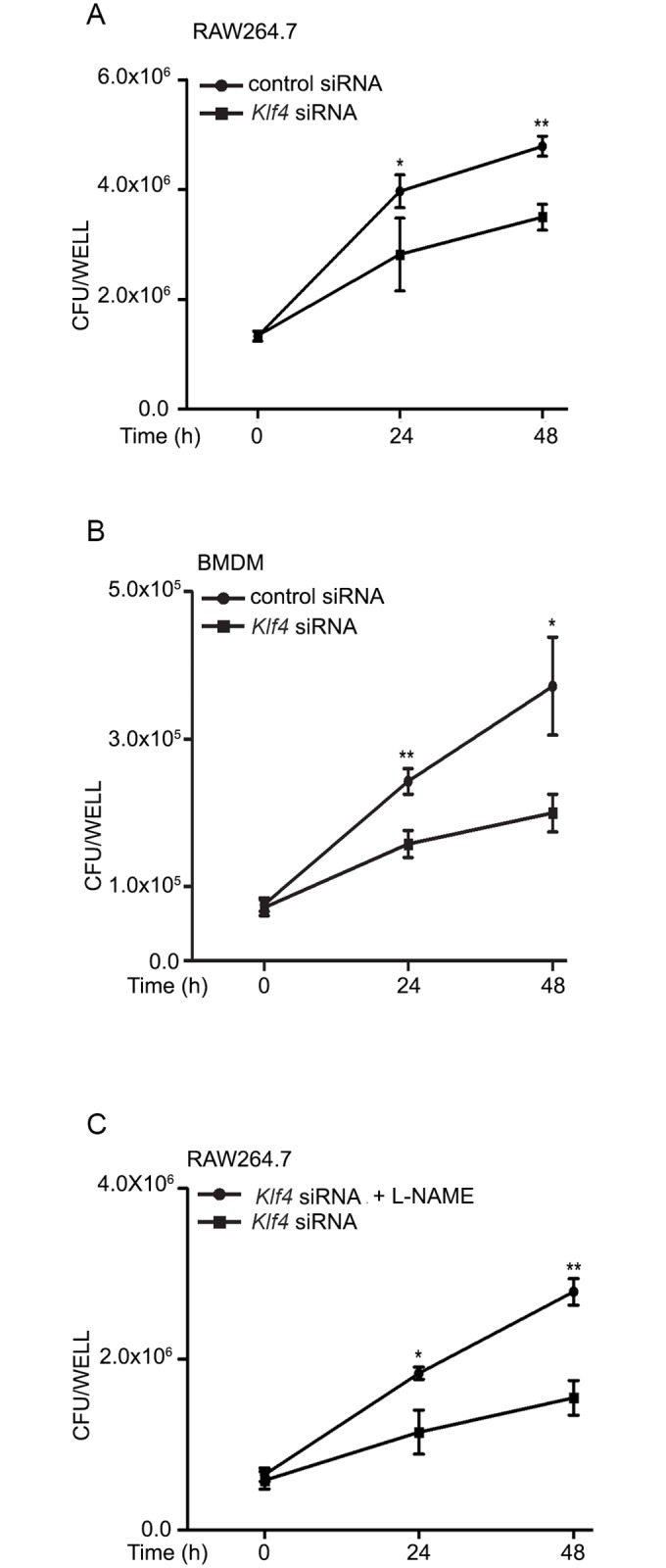
KLF4 regulates bacterial survival in macrophages. RAW264.7 cells (A, C) or BMDMs (B) were transfected with control siRNA or *Klf4* siRNA. After 48 h, cells were infected with Mtb (A,B) or pretreated with L-NAME (C) prior to infection with Mtb. Bacterial counts were determined at different time points post-infection. Results are means ± SEM (n = 3). *p< 0.05; ** *p<*0.01.

M1 macrophages are characterized by the production of reactive oxygen species (ROS) and reactive nitrogen species (RNS) which are key to the elimination of intracellular pathogens. M2 macrophages, on the other hand, produce anti-inflammatory cytokines such as IL-10, as well as arginase 1 (Arg1) which competes with iNOS for the substrate arginine. In the light of reports that KLF4 tilts the balance towards M2 polarization [10], we tested the role of KLF4 in regulating iNOS and arginase activities. Silencing of *Klf4* (Fig 4A) repressed Mtb-induced arginase activity (Fig 4B and 4C) while augmenting Mtb-induced nitrite production (Fig 4D and 4E) and the expression of iNOS (Fig 4F), supporting the view that KLF4 is critical in regulating the iNOS/arginase balance during Mtb infection. In harmony with these results, transfection of cells with miR-26a mimic prior to infection, repressed arginase activity (Fig 4G and 4H) while augmenting nitrite production (Fig 4I and 4J) and iNOS expression (S4A Fig). Conversely, miR-26a inhibitor repressed Mtb-induced nitrite production (Fig 4K) but augmented arginase activity (Fig 4L). The role of miR-26a in transcriptional regulation of the iNOS/arginase balance was further strengthened by our observations that Mtb-induced iNOS promoter activity was enhanced in cells transfected with a miR-26a mimic, whereas arginase promoter activity was inhibited (S4A and S4B Fig). miR-26a therefore emerged as a novel miRNA regulating M1/M2 polarization during Mtb infection. The role of KLF4 in allowing the pathogen to establish itself in its intracellular niche was further supported by our observations that silencing of *Klf4* attenuated bacterial counts in macrophages (Fig 5A and 5B), in line with the effect of transfection of the miR-26a mimic on bacterial CFUs. In order to elucidate the role of NO in the KLF4- or miR-26a-dependent control of bacterial CFUs, we transfected cells with *Klf4* siRNA prior to infection. Following infection, cells were incubated with L-NAME (an inhibitor of iNOS) and bacterial counts were determined at different time points. Under conditions of *Klf4* knockdown, bacterial counts were higher in infected cells when the cells were treated with L-NAME after infection, compared to cells that were not treated with L-NAME (Fig 5C), suggesting that KLF4 regulates bacterial survival at least in part through regulating iNOS (Fig 4E) and NO (Fig 4F) levels.

### Overexpression of miR-26a augments autophagy in Mtb-infected macrophages

**Fig 6:**
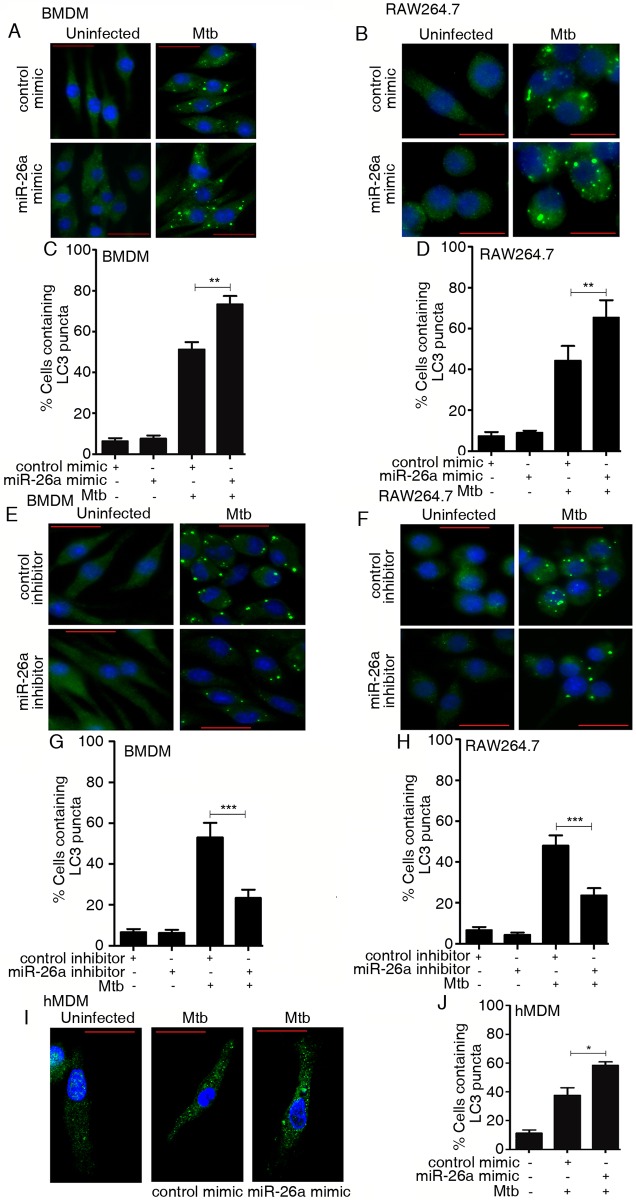
miR-26a regulates autophagy in Mtb-infected macrophages. BMDMs (A,E) or RAW264.7 (B,F) or hMDMs (I) were transfected with control mimic or miR-26a mimic (A,B, I); or control inhibitor or miR-26a inhibitor (E,F) prior to infection with Mtb. After 6 h, the cells were fixed and incubated with LC3 antibody, followed by staining with Alexa-488-conjugated secondary antibody. LC3 puncta (green) formation was detected by confocal fluorescence microscopy. The experiments were done in triplicate and at least 100 cells were counted for each condition. Results obtained for panels A, B, E, F and I are represented in panels C, D, G, H and J respectively. The data are representative of three independent experiments in RAW264.7 and two independent experiments in BMDMs and hMDMs. * *p<*0.05; ** *p<*0.01; *** *p<*0.001. Bar = 20 μm.

Host-directed molecules which redirect macrophage signaling towards activation of the xenophagic pathway during Mtb infection could offer the means of complementing Mtb-directed drugs towards improved therapeutic regimens. In an effort to understand the role of miRNA-transcription factor crosstalk in regulating Mtb-induced autophagy in macrophages, we analyzed the role of miR-26a in this process. Transfection of macrophages with miR-26a mimic prior to infection, augmented the Mtb-induced conversion of LC3-I to LC3-II as assessed by Western blotting (S5A and S5B Fig). In addition, *Map1lc3b* levels were also augmented in these cells (S5D Fig). Fluorescence microscopy confirmed the increased formation of LC3 puncta in infected murine (Fig 6A–6D) and human (Fig 6I and 6J) macrophages. At the same time, a miR-26a inhibitor, repressed the formation of LC3 puncta associated with Mtb infection (Fig 6E–6H), suggesting that the infection-associated downregulation of miR-26a represses autophagy.

### *Klf4* silencing augments autophagy in Mtb-infected macrophages

**Fig 7:**
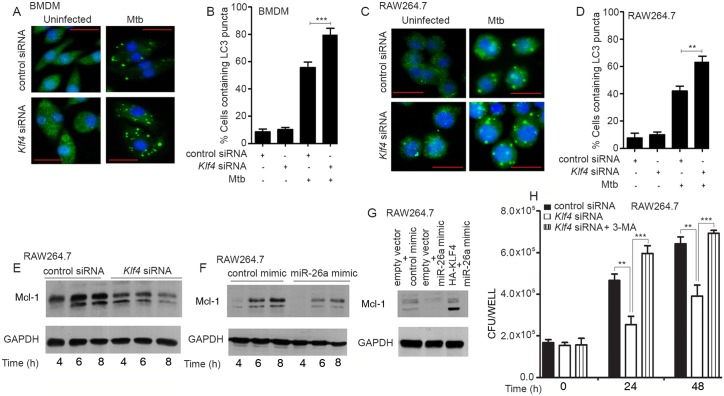
KLF4 regulates autophagy in Mtb-infected macrophages. (A, B) BMDMs or RAW264.7 (C,D) were transfected with control siRNA or *Klf4* siRNA prior to infection with Mtb. After 6 h, the cells were fixed and incubated with LC3 antibody, followed by staining with Alexa-488-conjugated secondary antibody. LC3 puncta (green) formation was detected by fluorescence microscopy. The experiments were done in triplicate and at least 100 cells were counted for each condition. The data are representative of results obtained in three independent experiments in RAW264.7 and two independent experiments in BMDMs. ** *p<*0.01; *** *p<*0.001. (E-G) Mcl-1 levels were analyzed after infection of RAW264.7 with Mtb. Cells were treated with control siRNA or *Klf4* siRNA (E) or control mimic or miR-26a mimic (F) or combinations of control mimic, miR-26a mimic, empty vector or HA-KLF4 plasmid as indicated (G), prior to infection. Blots are representative of the results obtained in two independent experiments. (H) Cells were treated with either control siRNA or *Klf4* siRNA for 48 h. Where indicated, cells were treated with 3-MA prior to infection. Bacterial counts were determined at the indicated time points. Results represent the means ± SEM (n = 3) ** *p<*0.01; *** *p<*0.001. Bar = 20 μm.

The role of the miR-26a/KLF4 axis in regulating autophagy was further evaluated by silencing *Klf4* prior to infection. The conversion of LC3-I to LC3-II (S5C Fig), levels of *Map1lc3b* (S5E Fig), as well as the formation of LC3 puncta (Fig 7A–7D) were enhanced upon *Klf4* knockdown. Further, the enhancement of LC3 puncta formation in the presence of a miR-26a mimic, was reversed when KLF4 was concomitantly overexpressed (S5F and S5G Fig). These results suggested that miR-26a/KLF4 signaling regulates autophagy during Mtb infection. We asked the question whether the miR-26a/KLF4 axis influences autophagy by transcriptional modulation of a regulator of autophagy. Mcl-1 is a transcriptional target of KLF4 [36]. Further, Mcl-1 has been reported by us to be a negative regulator of autophagy in Mtb-infected macrophages [32]. We therefore tested the role of miR-26a/KLF4 in regulating the levels of Mcl-1. Knockdown of *Klf4* attenuated the expression of Mcl-1 in Mtb-infected macrophages (Fig 7E). As expected, transfection of cells with a miR-26a mimic prior to infection, also attenuated the expression of Mcl-1 (Fig 7F). When cells were transfected with a miR-26a mimic along with a plasmid expressing KLF4 prior to infection, the levels of Mcl-1 increased compared to cells transfected with miR-26a mimic alone (Fig 7G). These observations confirmed that miR-26a attenuates, and KLF4 activates Mcl-1 expression. Taken together, these results suggested that the miR-26a/KLF4 axis regulates autophagy is at least in part due to its role in regulating the levels of Mcl-1. In order to test whether the regulation of autophagy by KLF4 is associated with altered bacterial survival in macrophages, bacterial CFUs were determined in macrophages infected with Mtb in the absence of presence of the autophagy inhibitor 3-methyladenine (3-MA). The repression in bacterial survival following knockdown of *Klf4* could be rescued by 3-MA (Fig 7H), suggesting that KLF4 enhances bacterial survival at least in part through inhibition of autophagy.

### miR-26a/KLF4 signaling regulates trafficking of Mtb to lysosomes

**Fig 8:**
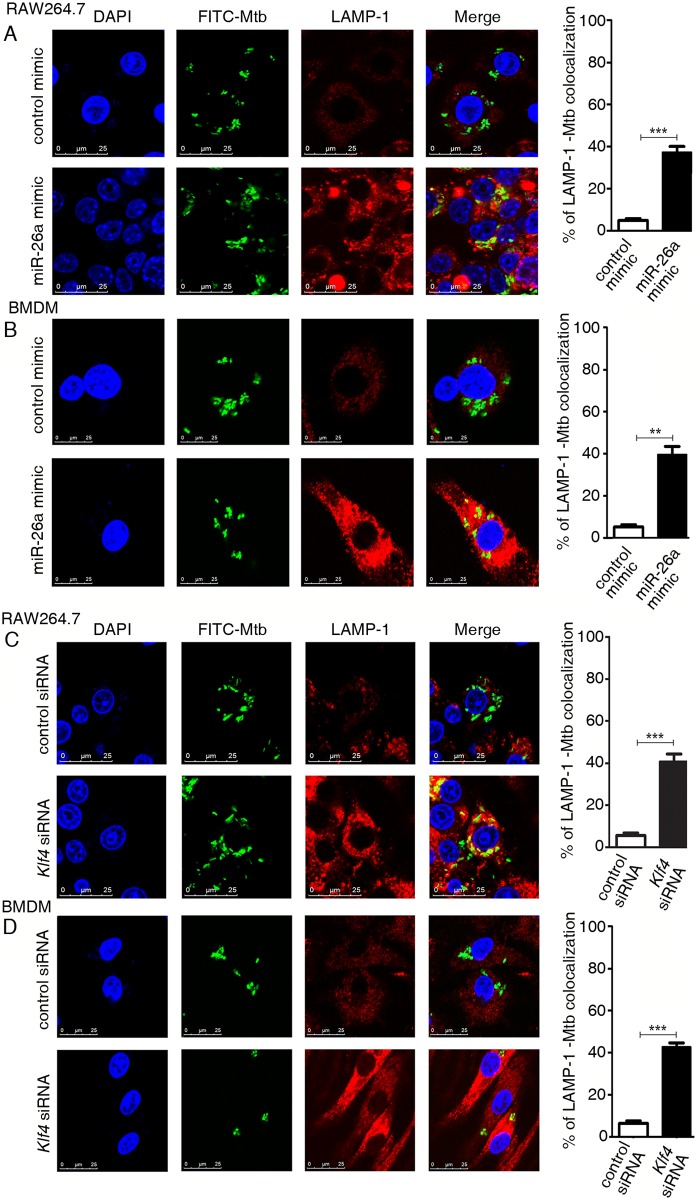
miR-26a and KLF4 regulate trafficking of Mtb to macrophages. (A,B) RAW264.7 (A) or BMDMs (B) were transfected with control mimic or miR-26a mimic for 24 h (C,D) RAW264.7 (C) or BMDMs (D) were transfected with control siRNA or *Klf4* siRNA for 48 h. Cells were infected with FITC-labelled Mtb (green). After 24 h, cells were fixed and stained with LAMP1 antibody and Alexa-546 conjugated secondary antibody (red), and visualized by confocal microscopy. Nuclei were stained with DAPI. Colocalization of red and green fluorescence indicates that the mycobacteria reside in the lysosomal compartment. The panels on the right represent quantification of the results. The data are representative of three independent experiments in RAW264.7 and two independent experiments in BMDMs. ** *p<*0.01; *** *p<*0.001.

The reduced viability of intracellular Mtb associated with expression of a miR-26a mimic or knockdown of KLF4, prompted us to test whether Mtb trafficking to the lysosomes was enhanced under these conditions. RAW264.7 cells were infected with FITC-labeled Mtb and subsequent association of bacteria with the lysosomes was monitored by fluorescence microscopy using LAMP1 as a lysosomal marker. In cells transfected with miR-26a mimic, significantly higher numbers of intracellular bacteria colocalized with lysosomes (Fig 8A and 8B). The same was true when KLF4 was knocked down (Fig 8C and 8D). Further, Mtb-LAMP1 colocalization observed in the presence of a miR-26a mimic, was reversed when KLF4 was concomitantly overexpressed (S6 Fig). This suggested that the concomitant downregulation of miR-26a and upregulation of KLF4 offers a survival advantage to Mtb by inhibiting trafficking of Mtb to lysosomes.

### C/EBPβ is expressed during Mtb infection and augments arginase activity

**Fig 9:**
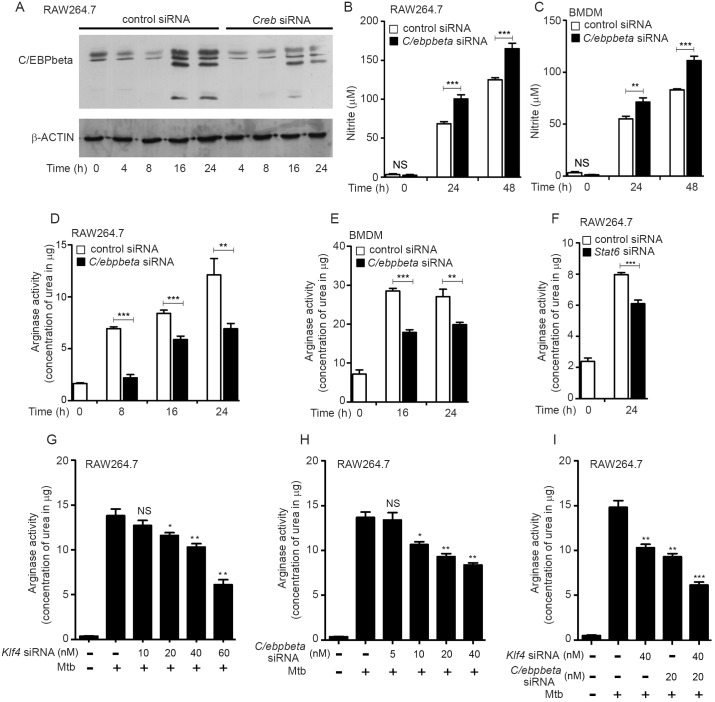
CREB-activated C/EBPβ regulates the arginase/NO balance during Mtb infection. (A) RAW264.7 cells were transfected with control siRNA or *Creb* siRNA for 48 h prior to infection with Mtb. Cells were lysed at different time points and lysates were analyzed by Western blotting for C/EBPβ. The blot is representative of the results obtained in three independent experiments. (B-F) Cells were transfected with siRNAs for 48 h, followed by infection with Mtb for different periods of time as indicated. Supernatants were analyzed for nitrite production (B,C); or arginase activity was assayed from the lysates (D-F). (G-I) Cells were transfected with *Klf4* or *C/ebpbeta*siRNA either alone (G, H) or in combination (I) at the doses indicated, for 48 h prior to infection with Mtb for 24 h. Arginase activity was assayed from the lysates. Data represent the means ± SEM (n = 3 for panels B, D, F-I; n = 2 for panels C and D). * *p<*0.05, ** *p<*0.01; *** *p<*0.001 (comparisons with uninfected cells); NS = not significant.

**Fig 10:**
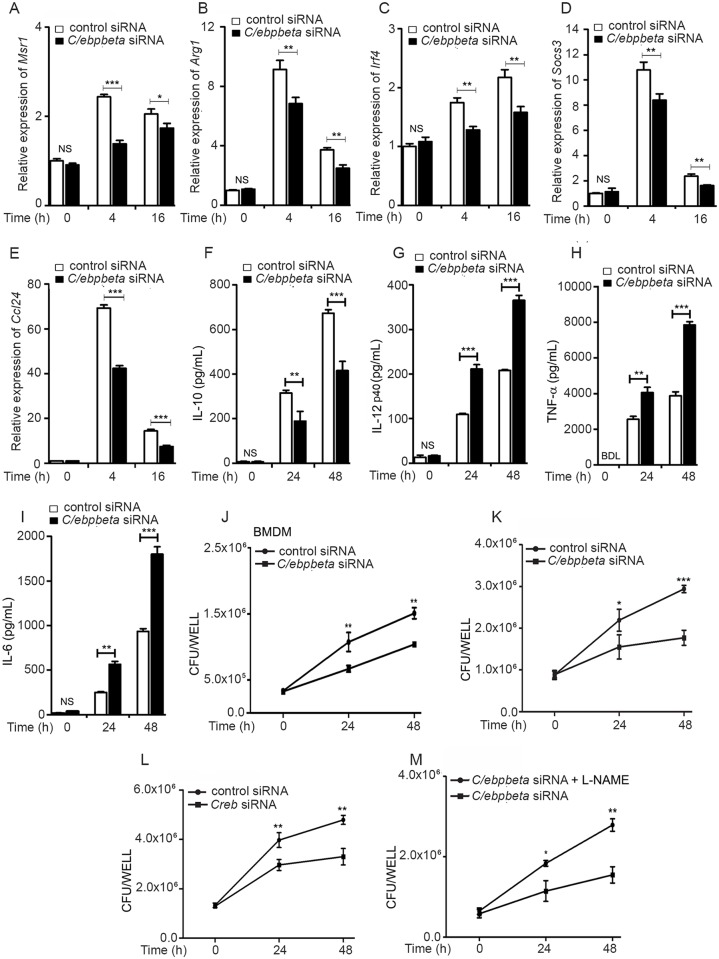
C/EBPβ regulates M1, M2 markers in Mtb-infected macrophages as well as the survival of Mtb in macrophages. Cells were transfected with control siRNA or *C/ebpbeta* siRNA (A-K; M); or with control siRNA or *Creb*siRNA (L) for 48 h prior to infection. (A-I) After infection of RAW264.7 cells with Mtb, relative expression of various M2 or M1 markerswas measured by qRT-PCR (A-E) or cytokine release was measured by ELISA (F-I) in the supernatants at different time points. (J-M) BMDMs (J) or RAW264.7 (K,L, M) were transfected as indicated, followed by infection with Mtb (K,L). In panel M, cells were treated with L-NAME before infection. Bacterial counts were determined at different time points. Data represent the means ± SEM (n = 3 for panels A-I, K-M; n = 2 for panel J). *p< 0.05; ** *p<*0.01; *** *p<*0.001. NS, not significant; BDL, below detection limit.

C/EBPβ plays a central role in regulating the M1/M2 phenotype of macrophages [16]. In partnership with NF-κB it directs macrophages towards M1 polarization with the release of proinflammatory cytokines, whereas in partnership with STAT6 it directs macrophages towards M2 polarization and the production of anti-inflammatory cytokines and M2-specific surface markers [34]. *C/ebpbeta* was upregulated in Mtb-infected macrophages (Fig 1B) and in the tissues of infected mice (S7 Fig). We tested whether CREB regulates C/EBPβ during Mtb infection. Knockdown of *Creb* (S8A Fig), attenuated the expression of C/EBPβ during Mtb infection (Fig 9A). Silencing of *C/ebpbeta* (S8B Fig) was associated with increased levels of nitrite (Fig 9B and 9C) but decreased arginase activity (Fig 9D and 9E). STAT6 is reported to co-operate with C/EBP-β to regulate arginase expression. Knockdown of *Stat6* (S8C Fig) attenuated Mtb-induced arginase expression (Fig 9F). It therefore appeared likely that C/EBPβ cooperates with STAT6 to activate arginase expression during Mtb infection. Knockdown of either *Klf4* or*C/ebpbeta* inhibited arginase activity in infected macrophages in a dose-dependent manner (Fig 9G and 9H), whereas knockdown of both had a synergistic effect on inhibition of arginase activity in infected macrophages (Fig 9I). C/EBPβ reportedly activates M2 markers but represses M1 markers. During Mtb infection, we observed transcriptional activation of the M2-specific markers *Msr1*, *Arg1*, *Socs3*, *Irf4*, *Ccl24* and IL-10(Fig 10A–10F) and *Ccl17* (S8D Fig). Knockdown of *C/ebpbeta* repressed the levels of all the aforesaid markers. At the same time, the production of the M1 markers IL12p40, TNF-α and IL-6 during infection, was augmented by knockdown of *C/ebpbeta* (Fig 10G–10I). These results strengthened the argument that C/EBPβ tilts the balance towards M2 polarization during Mtb infection.

### CREB and C/EBPβ influence the survival of Mtb in macrophages

We argued that since CREB-C/EBPβ signaling was associated with enhanced arginase activity, reduced NO production and a swing towards M2 polarization, this would likely facilitate survival of Mtb in macrophages. In support of this, we observed that knockdown of either *Creb* or *C/ebpbeta* was associated with a reduction of bacterial CFUs in macrophages (Fig 10J–10L). That C/EBPβ acts, at least in part by regulating NO levels, was borne out by our observation that bacterial counts under conditions of knockdown of *C/ebpbeta*, were higher in the presence of L-NAME than in its absence (Fig 10M).

## Discussion

During the early phases of infection, Mtb employs mechanisms to escape immune surveillance and to elicit a macrophage response normally associated with the resolution of inflammation. To date we lack comprehensive knowledge of the key regulatory pathways that are exploited by the bacterium to establish infection during the early stages of its intracellular lifestyle.

Transcription factors (TFs), and posttranscriptional regulators regulate M1/M2 macrophage polarization. TFs which regulate M1/M2 polarization and inflammatory signaling have been historically linked to the immune response to infection. During the early stages of infection, M1 polarization is associated with the control of Mtb infection [37]. In spite of the release of a battery of cytokines and chemokines by the macrophage, under certain circumstances Mtb overcomes the host arsenal and establishes infection. Of note, the transcriptional and post-transcriptional reprogramming of the host macrophage during Mtb infection, remain poorly understood. The present study uncovers important host cell determinants involving the miRNA, miR-26a and the transcription factors, KLF4, CREB and C/EBPβ that are associated with mycobacteria-induced M2 polarization, and repression of autophagy as well as lysosomal trafficking of Mtb, leading to enhanced survival of the bacterium.

TB susceptibility parallels with elevated levels of type-2 inflammatory signals [38]. M2 macrophages are abundant in granulomas from tuberculosis patients [39]. We therefore rationalized that it is important to understand the regulatory mechanisms involved in M2 polarization during Mtb infection. Making use of data of the Mtb-infected macrophage transcriptome (at 4 h and 24 h post-infection) available from our laboratory, we narrowed down on a set of transcription factors that are reportedly important players in M1/M2 macrophage polarization [34], namely KLF4, C/EBPβ and CREB. We observed transcriptional downregulation of KLF4 and upregulation of C/EBPβ in infected RAW264.7 cells. Interestingly, both these transcription factors were upregulated at the protein level. This suggested a post-transcriptional mechanism of upregulation of KLF4.

Decoupling of mRNA and protein expression observed in processes such as aging [40], depends on interactions between cis-acting sequence elements located on the target RNA and trans-acting factors such as RNA binding proteins and non-coding RNAs such as miRNAs. Posttranscriptional regulation is exerted by molecular crosstalk between *cis*-acting sequence elements located on the target RNA and *trans*-acting regulatory factors: RNA binding proteins (RBPs) and non-coding RNAs, such as miRNAs [41–43].

miRNAs play important roles in macrophage polarization [44]. We therefore hypothesized that miRNA-mediated regulation could be a possible mechanism for the discordant expression levels of KLF4 transcript and protein following Mtb infection. From our previously archived data, we searched for miRNAs which could potentially target KLF4 and which were also downregulated during infection. We narrowed down on miR-26a, one of the miRNAs that was downregulated in Mtb-infected macrophages. *Klf4* was subsequently identified as a bonafide miR-26a target. miRNA-mediated regulation therefore explains at least in part, the discordant expression of the KLF4 transcript and protein, although other mechanisms cannot be ruled out. The early upregulation of KLF4 in Mtb-infected macrophages, suggests that Mtb infection possibly impacts post-translational mechanisms of regulation of KLF4 as well. A post-transcriptional mechanism of upregulation of KLF4 linked to the downregulation of cellular miR-145 has been reported during infection of keratinocytes with human papillomavirus [45]. KLF4 can act as either a positive or negative regulator of transcription when bound to promoters, thereby regulating divergent functions depending on the cell type and the context. In connection with Mtb infection, KLF4 is transcriptionally upregulated during infection of Rhesus monkey BMDMs with Mtb subjected to hypoxic stress, whereas it is transcriptionally downregulated during infection with Mtb grown under normoxic conditions [46] suggesting that KLF4 potentially regulates the immune response to infection. Ghorpade *et al*. [47] have established a link between KLF4 and downregulation of MHC class II-dependent antigen presentation in *M*. *bovis* BCG- infected macrophages. Our studies show for the first time in the context of mycobacterial infection, that the miR-26a/KLF4 signaling axis is a determinant of the M1/M2 macrophage phenotype. The repression of miR-26a during infection is associated with enhanced KLF4 which skews the balance between arginase and iNOS towards the former. We further observed that increased miR-26a expression or knockdown of KLF4 significantly attenuates survival of Mtb in macrophages, suggesting that this signaling axis is an important player in the innate immune response of macrophages. The effect of knockdown of KLF4 could be partly offset by the presence of L-NAME, an inhibitor of iNOS, strengthening the contention that regulation of iNOS levels by miR-26a/KLF4 plays a role in bacterial survival in macrophages. It may be mentioned that a study by Ni *et al*. [48] has reported increased miR-26a levels in Mtb-infected human macrophages. However, the conditions of the study by Ni *et al*. [48] were substantially different from the present one. Significantly, Ni *et al*. used Mtb grown on plates rather than early log phase cultures of Mtb used here for infection. The multiplicities and time of infection used for analysis of miRNAs, also differed from those of the present study. In this study, hMDMs were differentiated in FBS and antibiotics, whereas Ni *et al*. used hMDMs cultured in human serum without antibiotics. One or more of these factors could contribute to the differences in observed levels of miR-26a. In this context it is pertinent to mention that decreased expression of miR-26a has been observed in peripheral blood from tuberculosis patients [49]. We have also observed decreased levels of miR-26a in mice infected with aerosolized Mtb.

Autophagy is an important arm of the macrophage defense machinery against an invading pathogen. Induction of autophagy overcomes the phagosome maturation block associated with Mtb infection, and facilitates trafficking of Mtb to lysosomes [50, 51]. During active tuberculosis, autophagy suppresses bacterial burden [52]. Considering that miR-26a and KLF4 regulated bacterial survival in macrophages, we tested their effects on Mtb-triggered autophagy. Transfection of a miR-26a mimic or knockdown of KLF4 were both associated with enhanced autophagy as evidenced by the conversion of LC3-I to LC3-II or the formation of LC3 puncta. Searching for regulators of autophagy which are transcriptionally activated by KLF4, we narrowed down on Mcl-1, which we have previously documented as a negative regulator of autophagy during Mtb infection [32]. We observed miR-26a- and KLF4-dependent modulation of Mcl-1 in Mtb-infected macrophages. These observations suggested that the miR-26a/KLF4/Mcl-1 axis was involved in regulating autophagy. Autophagy promotes the clearance of Mtb by enhancing trafficking of Mtb to lysosomes. In this report we show that forced expression of a miR-26a mimic or knockdown of KLF4 promotes trafficking of Mtb to lysosomes.

The second TF that we focused on, C/EBPβ, is a regulator of M1/M2 polarization of macrophages. It is expressed at basal levels in M1 macrophages but upregulated for M2 activation [53]. Deletion of two CREB-binding sites from the CEBPβ promoter abrogates its induction upon macrophage activation [16], linking the TF CREB with CEBPβ.

CREB-C/EBPβ signaling has been linked to induction of the M2-specific genes *Msr1*, *Il10*, *II13ra*, and *Arg1*. Tilt towards M2 polarization weakens the defense of the host against Mtb infection [54, 55]. In this report we show that CREB-C/EBPβ signaling is central to the survival of Mtb in macrophages. CREB-dependent C/EBPβ expression showed a temporal increase in Mtb-infected macrophages. In macrophages, miR-155 targets C/EBPβ[56]. However, this axis is unlikely to play a role in the present context, considering that miR-155 is upregulated during Mtb infection [57]. Importantly, knockdown of C/EBPβ was associated with an increased production of iNOS and NO and a concomitant decreased activity of arginase. Considering the role of C/EBPβ on M1/M2 polarization, we tested the expression of markers of M1 and M2 macrophages following knock down of C/EBPβ. M2 markers were repressed whereas the M1 markers IL-6, TNF-α and IL-12 p40 were enhanced when C/EBPβ was knocked down prior to infection, suggesting that C/EBPβ regulates the M1/M2 balance in infected macrophages.

miR-26a has been reported to target checkpoint kinases to regulate the cell cycle during genotoxic stress [58] and to target the histone methyltransferase Enhancer of Zeste homology 2 during myogenesis [59]. Here we present a novel role of miR-26a in innate immunity. Differential regulation of miR-26a and KLF4 regulates autophagy and trafficking of Mtb to lysosomes. In addition, C/EBPβ regulates the M1/M2 balance. The link between miR-26a/KLF4 and C/EBPβ has not been investigated in detail in this study. Kapoor *et al*. [11] have demonstrated that KLF4 induces MCP-1–induced protein (*Mcpip*), which in turn activates the transcription of C*/ebpb*. We therefore speculate that MCPIP could be the missing link between KLF4 and C/EBPβ.

**Fig 11:**
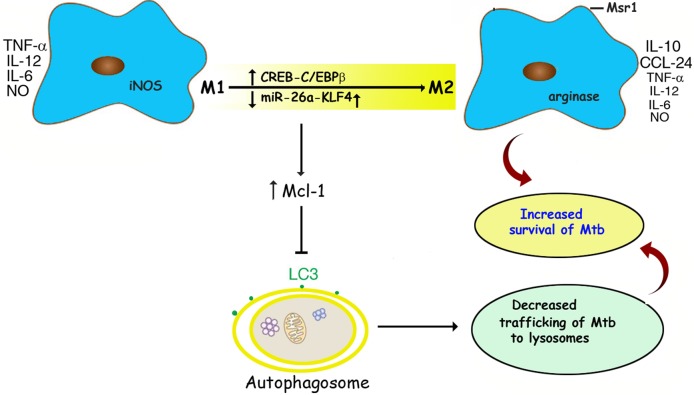
Schematic representation of the post-transcriptional and transcriptional regulation of M1/M2 polarization and autophagy in Mtb-infected macrophages. Mtb infection is associated with the downregulation of miR-26a which in turn upregulates its target KLF4; and CREB-mediated transcriptional upregulation of C*/ebpbeta*. This is associated with decreased expression of M1 markers such as TNF-α, IL-12, IL-6, and iNOS with a concomitant increase in the expression of M2 markers such as IL-10, CCL24, arginase and Msr1. Further, upregulation of KLF4 favours increased expression of Mcl1 which in turn inhibits autophagosome formation and consequently, lysosomal trafficking of Mtb. Mtb infection thus tilts the innate immune response of the host towards pathways that favor the survival of the bacterium.

Our observations provide a link towards understanding how Mtb exploits the immune response of macrophages to successfully create its own intracellular niche (Fig 11). Mechanistically, we establish that miRNA-TF crosstalk allows the pathogen to survive during infection. This knowledge may in the long run help in the development of host-directed therapies targeting the microRNA and/or the specific TFs described in this study.

## Methods

### Reagents, antibodies and plasmids

For Northern blotting, digoxigenin (DIG)-labelled probes for miR-26a and U6 were from Exiqon. Lipofectamine 2000, JET PEI and Dharmafect II transfection reagents were purchased from Invitrogen, Polyplus and Thermo Scientific respectively. Macrophage colony stimulating factor (M-CSF) was purchased from Prospec. miR-26a mimic, miR-26a inhibitor, control inhibitor and control mimic were purchased from Ambion. siRNA against *Klf4*, *Creb* and *Stat6*, and control siRNA were purchased from Eurogentec. N-nitro-L-arginine methyl ester (L-NAME), Phorbol 12-myristate 13-acetate (PMA), 3-methyladenine were from Sigma Chemical Co. *C/ebpbeta* siRNA was purchased from Ambion and used at a final concentration of 20 nM.

Antibodies were sourced as follows. C/EBPβ (sc-150) and GAPDH (sc-25778) were from Santa Cruz Biotechnology, LC3 (2775) was from Cell Signaling Technology (for Western blotting) or from MBL (PM036) (for immunofluorescence), KLF4 (ab129473), CREB (ab178322), LAMP1 (Ab 25245) and iNOS (ab3523) were from Abcam. β-actin (A2547) antibody was from Sigma Chemical Co. The HRP tagged secondary antibodies were from Cell Signaling Technology. Alexa-488 and Alexa-546 conjugated secondary antibody, Griess reagent and Calcein AM (Cat. No.-C3100MP) were from Invitrogen (Molecular Probes). The following plasmids were sourced from Addgene: pGL3-mArg1 promoter/enhancer (#34571), pGL2-NOS2 promoter (#19296), and pcDNA3.1-HA-KLF4-FL (34593).

### Mycobacterial strains and growth conditions

Mtb H37Rv was grown in Middlebrook 7H9 medium with 10% ADC (Becton Dickinson) and 0.05% Tween 80. For infections, cultures with an OD_600_ around 0.2 were pelleted and resuspended in DMEM with 10% FBS.

### Cell culture and infection

RAW264.7 murine macrophage-like cells were obtained from the National Cell Science Centre (NCCS), Pune, India and grown in RPMI-1640 medium supplemented with 10% FBS, penicillin/streptomycin and 5 mM L-glutamine (Life Technologies), at 37°C with 5% CO_2_. HEK293 cells were obtained from NCCS and grown in MEM supplemented with 10% FBS, penicillin/streptomycin at 37°C with 5% CO_2_. THP-1 cells were differentiated in RPMI-1640 medium supplemented with 10% FBS, penicillin/streptomycin and PMA (50 nM) for 24h followed by incubation in fresh medium for another 24h. BMDMs were prepared from bone marrow obtained from the femurs and tibia of Balb/C mice by culturing in IMDM containing 10% FBS, penicillin/streptomycin and M-CSF at 37°C with 5% CO_2_ for 6–8 days for their maturation. hMDMs were prepared from blood obtained from healthy volunteers. Monocytes were differentiated in RPMI supplemented with 10% FBS, penicillin/streptomycin for 6 to 8 days. Mtb infections were carried out for 4 h at an MOI of 10, followed by washing and incubating cells in gentamycin containing medium for 2 h to remove adhered bacteria. Cells were further incubated in DMEM with 10% FBS for the indicated time periods.

### RNA isolation and Northern blotting

Total RNA was isolated using the mirVana miRNA isolation kit (Ambion) according to the manufacturer’s instructions. Northern blotting was carried out using DIG-labeled LNA Probes (from EXIQON) as described previously [30]. RNA-containing membranes were washed with DIG-WASH buffer followed by incubation with CSPD (Roche) at 37°C for 10 min. Membranes were exposed to X-ray film and developed. Membranes were reprobed with DIG-labelled U6 probe (Exiqon, Product No. 99002–15) as a loading control.

### RT-PCR

The RevertAid First Strand cDNA Synthesis Kit (Fermentas) was used for preparing cDNA. The expression levels of pri-miR-26a-1 and pri-miR-26a-2 were detected by semi-quantitative PCR using ExPrimeTaq DNA polymerase. PCR was carried out for 26 cycles and *Gapdh* was used as loading control. For quantitation of other mRNAs, real time PCR was carried out in triplicate using SYBR Green based real time PCR. Details of primers used are given in S2 Table.

### Western blotting

This was performed as described previously [30]. Cells were lysed in lysis buffer (Cell Signaling technology). Proteins were separated by SDS-PAGE and transferred to PVDF membranes. Membranes were incubated in 5% non-fat dry milk in 1X Tris-buffered saline containing Tween 20 (TBST) for 1 h at room temperature, washed with TBST and incubated overnight at 4°C with primary antibodies followed by washing and incubation with secondary antibodies in 5% non-fat dry milk. Blots were developed using Lumiglo (Cell Signaling Technology).

### Generation of KLF4 3’UTR reporter constructs and luciferase reporter assay

The KFL4 3′UTR containing the seed region for miR-26a was amplified using mouse genomic DNA as template and the sense and antisense primers 5′TCACTAGTATCCCACGTAGTGGATGTGA3′ (a) and 5′AACAAGCTTCTTATTTCTCACCTTGAGTATGC3′ (d) respectively. The PCR product was cloned in pMIR-Report between the asymmetric SpeI and HindIII sites. Site directed mutagenesis of the seed region in the KLF4 3′UTR was performed by overlap extension PCR using the primer pairs a (sense) and 5′TCCAATACCAAATCCACATGCCCGGACTTACA3′ (b,antisense); and 5′TGTAAGTCCGGGCATGTGGATTTGGTATTGGA3′ (sense, c) and d (antisense). The final PCR product was generated using the aforementioned products as templates and primers “a” and “d”, cloned in pMIR-Report and sequenced. For luciferase reporter assays, KLF4 3′UTR constructs were cotransfected with miR-26a mimic (or control mimic) and a β-galactosidase expressing construct in HEK293 cells using Lipofectamine 2000. After 24 h of transfection, cells were lysed and luciferase activity was measured using the Luciferase Reporter Assay System (Promega). Luciferase activity was normalised for transfection efficiency by assaying β-galactosidase activity.

### Immunoprecipitation of c-Myc-Ago2-containing RISC

The recruitment of KFL4 transcript to Ago2-complexes was determined as described [30]. Briefly, RAW264.7 cells were transfected with c-Myc-tagged Ago2 (a gift from Jidong Liu, Memorial Sloan-Kettering Cancer Center, NY) and miR-26a mimic or control mimic, allowed to grow for 24 h and lysed in cold lysis buffer (20 mM Tris-HCl (pH 7.5), 150 mM NaCl, 1mM Na_2_EDTA, 1 mM EGTA, 1% Triton X-100, 2.5 mM sodium pyrophosphate, 1 mM β-glycerophosphate, 1 mM Na_3_VO_4_, 1 μg/ml leupeptin, 100 units/ml RNasin Plus (Promega) supplemented with 1 mM PMSF and complete protease inhibitor cocktail (Roche) for 30 min at 4°C. Supernatants were incubated with anti-c-Myc-Agarose (Sigma) or protein A/G Plus-Agarose (Santa Cruz Biotechnology) that had been previously blocked in IP wash buffer (300 mM NaCl, 5 mM MgCl_2_, 50 mM Tris-HCl (pH 7.5), 0.1% Triton X-100) and calibrated in lysis buffer containing yeast t-RNA (Promega) for 3 h at 4°C with rotation. Immune complexes were washed successively with lysis buffer, IP wash buffer and cold PBS to remove residual detergent. RNA was prepared using the miRvana kit (Ambion) according to the manufacturer’s instructions. RNA samples were eluted from columns in 30 μl H_2_O and used for quantification of KLF4 by qRT-PCR. GAPDH was used for normalization.

### Arginase assay

Arginase activity was measured in macrophages by the method of Corraliza*et al*. [60]. Briefly, lysates were tested for arginine hydrolysis activity by incuation with L-arginine. The production of urea was quantified using α-isonitrosopropiophenone (ISPF). Details are provided in S1 Text.

### Nitric oxide measurement

NO release was assayed by measuring the production of nitrite using Griess reagent. Details are provided in S1 Text.

### Promoter activation assays

The *Arg1* or *iNOS* promoter fused to the firefly luciferase gene was cotransfected with control mimic or miR-26a mimic in RAW264.7 cellls along with a β-galactosidase expressing construct using Lipofectamine 2000. After 24 h of transfection, cells were infected with Mtb for different periods of time. Cells were lyzed and luciferase activity was measured using the luciferase assay kit (Sigma) according to the manufacturer’s instructions. Luciferase activity was normalized by measuring β-galactosidase activity using the β-galactosidase activity assay kit (Promega).

### Immunostaining and fluorescence microscopy

For immunostaining, cells were plated on cover slips in 24 well plates at a seeding density of 10^5^ cells/well a day before the experiment. After infections, cells were fixed, permeabilized and immunostained as described by Kumar *et al*. [33] before analysis by confocal microscopy (TCS SP8, Leica Microsystems, Germany).

### Bacterial CFU determinations

RAW264.7 cells or BMDMs were plated in 48 well plates. Cells were infected with Mtb at an MOI of 10. Infected cells were lysed, serial dilutions of homogenates were plated on Middlebrook 7H11 agar plates supplemented with 10% OADC and incubated at 37°C for 3–4 weeks. CFUs were calculated in triplicate using standard procedures.

### Cell viability assay

Viability of macrophages was quantitated using the calcein assay kit. Briefly, cells were washed with PBS thrice and incubated with calcein-AM (250 nM) in PBS for 30 min at 37°C to stain viable cells prior to analysis. Fluorescence was measured in a VICTOR^3^ plate reader (Perkin Elmer). Percent survival was calculated as a percentage of the fluorescence of untreated control cells.

### Infection of mice

Mice were purchased from the Central Drug Research Institute, Lucknow and were infected by aerosol inhalation of Mtb H37Rv at the National Jalma Institute for Leprosy and other Mycobacterial Diseases, Agra as described [30]. Effective dose of infection was confirmed by harvesting the lungs from mice 1 day after infection and determining colony-forming units. Infections were performed at a dose of about 100 cfu per mouse. Lungs, spleen and lymph nodes were isolated, homogenized in Trizol and total RNA was isolated.

### miRNA profiling

miRNA expression profiling was carried out by using qRT-PCR based TaqMan Low-Density Arrays (TLDA) in replicates at Life Technologies, Gurgaon, India. The results were analyzed using Data assist software v1.0. Data was normalized to snoRNA202, which showed least variation between C_T_ values of uninfected and infected samples. Fold induction was determined using the comparative C_T_ method.

### Statistical analyses

GraphPad Prism software was used for statistical analysis. Two groups were compared using 2-tailed Students *t*-test. The data were presented as means ± SEM and *p*-value less than 0.05 were considered significant.

### Ethics statement

Animal experiments were approved by the Institutional Animal Ethics Committees of the National JALMA Institute for Leprosy and Other Mycobacterial Diseases, Agra India (IAEC/JALMA/55/2015) and the Bose Institute, Kolkata, India (IAEC/BI/34/2015).

### Accession numbers

mRNA data sets can be found in the NCBI Geo Database under accession number GSE64427.

## Supporting information

S1 Fig Heat map of transcription factor expression during infection of RAW264.7 with Mtb (MOI, 10).228 transcription factors were selected using the TRANSFAC database. Out of these, 168 differentially regulated transcription factors were screened using Ingenuity Pathway Analysis. The microarray data sets used for generating this heat map can be found in the NCBI Geo Database under accession number GSE64427.
(TIF)

S2 Fig Viability of macrophages in the presence of miR-26a mimic or inhibitor, and Northern analysis of miR-26a expression in the organs of infected mice. The viability of macrophages transfected with either control or miR-26a mimic (A, C) or with either control or mir-26a inhibitor (B, D) was quantitated by the calcein assay. Data represent the means ± SEM (n = 3). (E-G) The expression of miR-26a was quantitated by Northern blotting as indicated in Fig 2J–2L. Densitometric analysis of the blots is presented in this figure. Each symbol represents one mouse. ***p<0.001. NS = not significant.
(TIF)

S3 Fig miR-26a is downregulated in Mtb-infected THP-1 macrophages and modulates KLF4 expression. (A) Differentiated THP-1 cells were infected with Mtb and the expression of miR-26a was analyzed by qRT-PCRusing U6 expression for normalization. Results are means ± SEM (n = 3). ***, *p*< 0.001. (B) PMA differentiated THP-1 macrophages were transfected with either control or miR-26a mimic and infected with Mtb for the indicated periods of time. Lysates were prepared and KLF4 expression was analyzed by Western blotting. The blot is representative of the results obtained in three independent experiments.
(TIF)

S4 Fig miR-26a modulates iNOS expression; and iNOS and arginase promoter activity in Mtb- infected macrophages. (A) RAW 264.7 cells were transfected with either control or miR-26a mimic, infected with Mtb for the indicated periods of time, lysates were prepared and iNOS expression was analyzed by Western blotting. The blot is representative of the results obtained in two independent experiments. Intensities of bands were measured by densitometric scanning. The fold change in iNOS was calculated with respect to uninfected cells. (B,C) RAW 264.7 cells were transfected with either control or miR26a mimic along with *iNOS* (B) or *Arg1* (C) promoter luciferase construct, followed by infection with Mtb for different periods of time. Cells were lysed and luciferase activity was measured. Results represent means ± SEM. **p<0.01.
(TIF)

S5 Fig miR-26a and KLF4 regulate autophagy in response to Mtb infection. RAW264.7 (A,C,D,E) or BMDMs (B) were transfected with miR-26a mimic or control mimic (A,B, D) or with *Klf4* siRNA (C, E); or with control mimic and empty vector or miR-26a mimic and empty vector or miR-26a +KLF4 expressing vector (F, G) prior to infection with Mtb. The conversion of LC3-I to LC3-II (A-C) or transcription of *Map1lc3b* (D,E) or the formation of LC3 puncta (F,G) was analyzed. Blots are representative of two separate experiments. For D, E and G, results represent means ± SEM, n = 3. * p< 0.05; ***p<0.001; NS: not significant.
(TIF)

S6 Fig Overexpression of KLF4 offsets the effect of miR-26a on trafficking of Mtb to macrophages. RAW264.7 cells or BMDMs were transfected with control mimic or miR-26a mimic for 24 h in combination with empty vector of KLF4 expressing plasmid as indicated. Cells were infected with FITC-labelled Mtb (green). After 24 h, cells were fixed and stained with LAMP1 antibody and Alexa-546 conjugated secondary antibody (red), and visualized by confocal microscopy. Nuclei were stained with DAPI. Colocalization of red and green fluorescence indicate that the mycobacteria reside in the lysosomal compartment. The panels on the right represent quantification of the results. The data represent three independent experiments in RAW264.7 and two independent experiments in BMDMs. *** *p<*0.001.
(TIF)

S7 Fig Analysis of *C/ebpbeta* in the organs of infected mice. The relative expression of *C/ebpbeta* was quantitated by qRT- PCR. Each symbol represents one mouse. *p<0.05.
(TIF)

S8 Fig Silencing of *Creb*, *C/ebpbeta* and *Stat6* and expression of *Ccl17* in Mtb-infected macrophages RAW264.7 cells were transfected with either control siRNA or *Creb* siRNA (A), *C/ebpbeta*siRNA (B) or *Stat6* siRNA (C) for 48 h. In the case of *C/ebpbeta*, transfected cells were infected with Mtb for the time periods indicated in panel B. Cells were lysed and immunoblotted with CREB (A), C/EBPβ (B) or STAT6 (C) antibody. Equal loading was confirmed by reprobing the blots with GAPDH β- actin antibody. Each blot is representative of the results obtained in three independent experiments. (D) RAW264.7 cells were transfected with either control or *C/ebpbeta* siRNA and infected with Mtb for different periods of time as indicated in the figure. RNA was isolated and the level of *Ccl17* was quantitated by RT-PCR. For (A-C), intensities of bands were measured by densitometric scanning. Results are indicated in the bar plots. Means ± SEM. (n = 3). ***p< 0.001
(TIF)

S1 Table Fold change of miRNAs that are downregulated during infection and are predicted to target KLF4. (DOCX)

S2 Table List of primers used for qRT-PCR in this study. (DOCX)

S1 Text (DOCX)
