# Supplementary figures and images for: MicroRNA 26a (miR-26a)/KLF4 and CREB-C/EBPβ regulate innate immune signaling, the polarization of macrophages and the trafficking of Mycobacterium tuberculosis to lysosomes during infection
Source: PLoS Pathog. 2017 May 30;13(5):e1006410. doi: 10.1371/journal.ppat.1006410 (PMC5466338; doi:10.1371/journal.ppat.1006410)

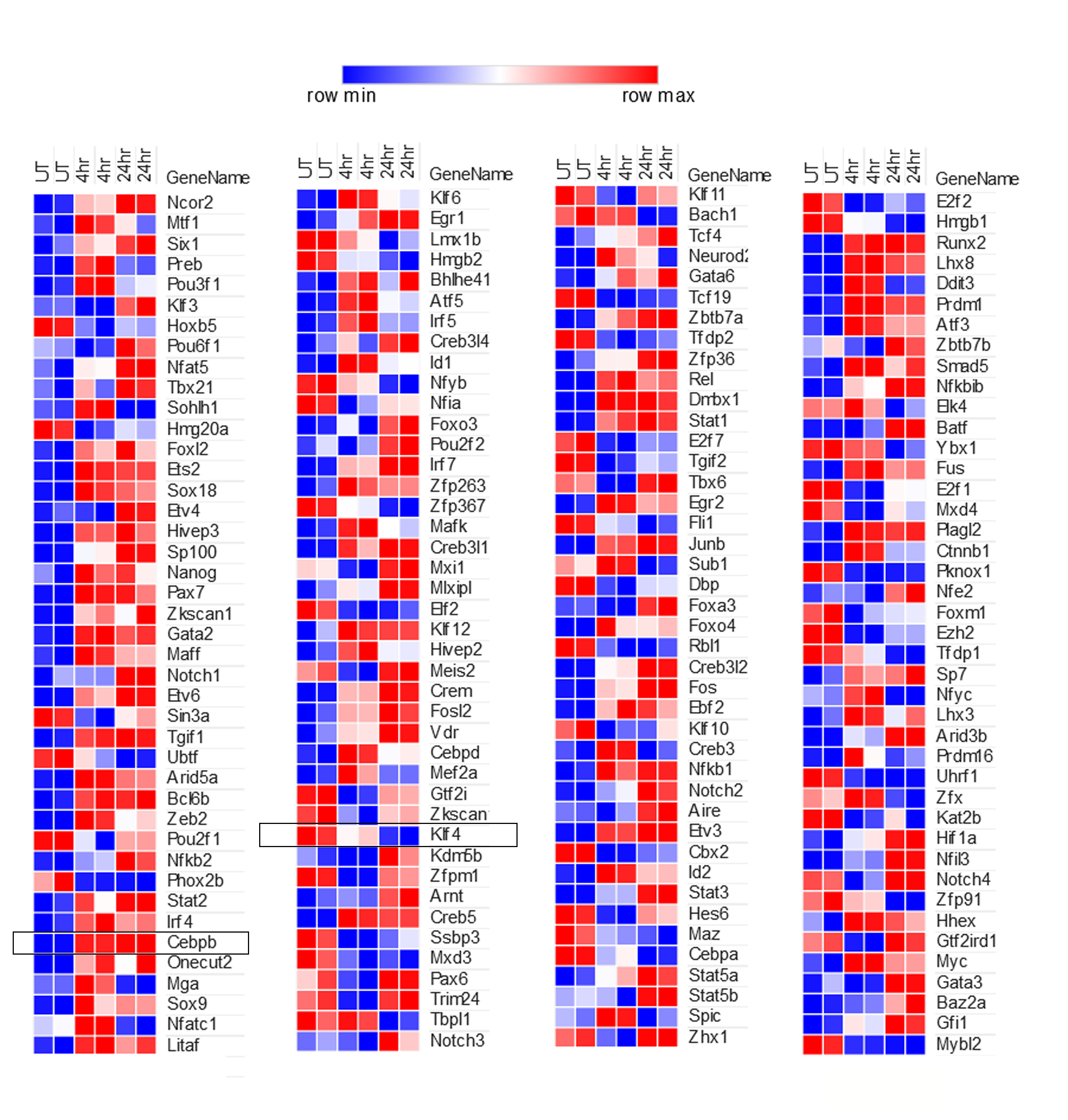

Supplement: S1 Fig — Out of these, 168 differentially regulated transcription factors were screened using Ingenuity Pathway Analysis. The microarray data sets used for generating this heat map can be found in the NCBI Geo Database under accession number GSE64427. (TIF) [file ppat.1006410.s001.tif]

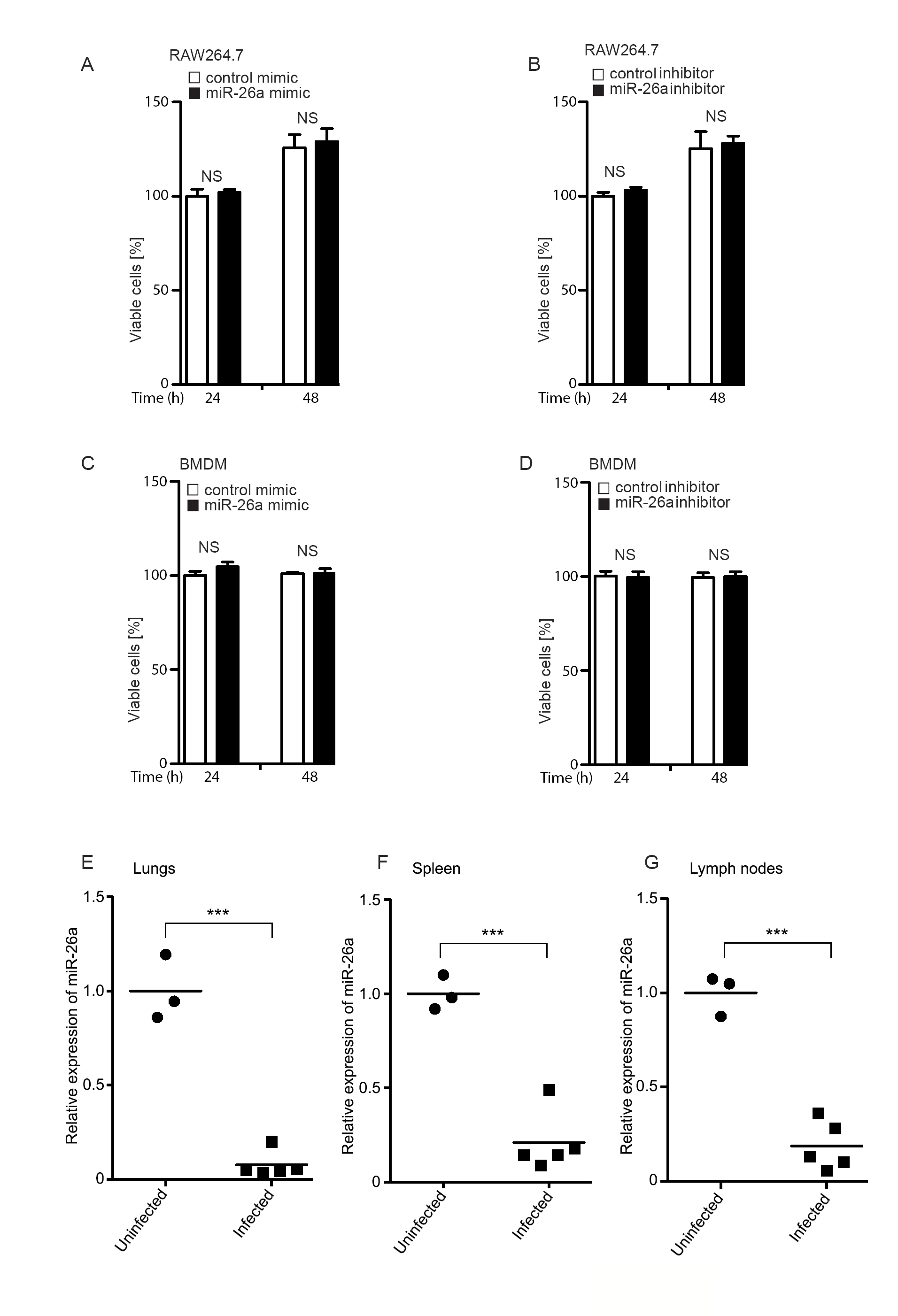

Supplement: S2 Fig — The viability of macrophages transfected with either control or miR-26a mimic (A, C) or with either control or mir-26a inhibitor (B, D) was quantitated by the calcein assay. Data represent the means ± SEM (n = 3). (E-G) The expression of miR-26a was quantitated by Northern blotting as indicated in Fig 2J–2L. Densitometric analysis of the blots is presented in this figure. Each symbol represents one mouse. ***p<0.001. NS = not significant. (TIF) [file ppat.1006410.s002.tif]

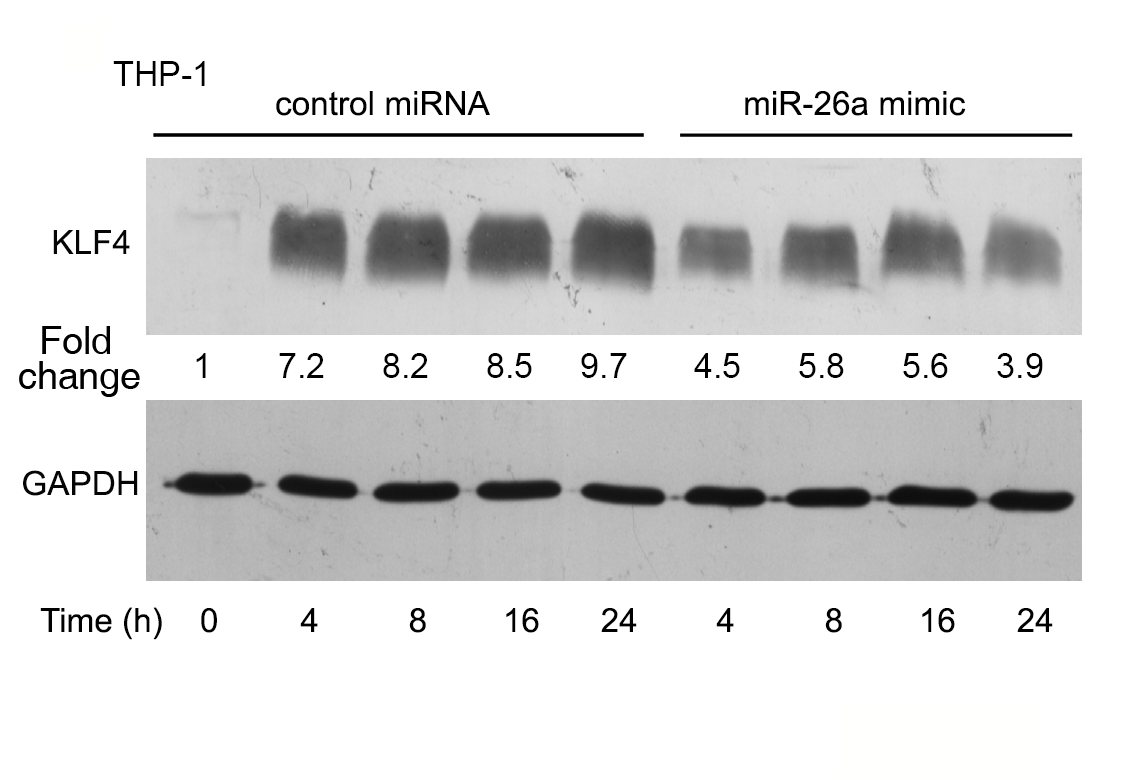

Supplement: S3 Fig — (A) Differentiated THP-1 cells were infected with Mtb and the expression of miR-26a was analyzed by qRT-PCRusing U6 expression for normalization. Results are means ± SEM (n = 3). ***, p< 0.001. (B) PMA differentiated THP-1 macrophages were transfected with either control or miR-26a mimic and infected with Mtb for the indicated periods of time. Lysates were prepared and KLF4 expression was analyzed by Western blotting. The blot is representative of the results obtained in three independent experiments. (TIF) [file ppat.1006410.s003.tif]

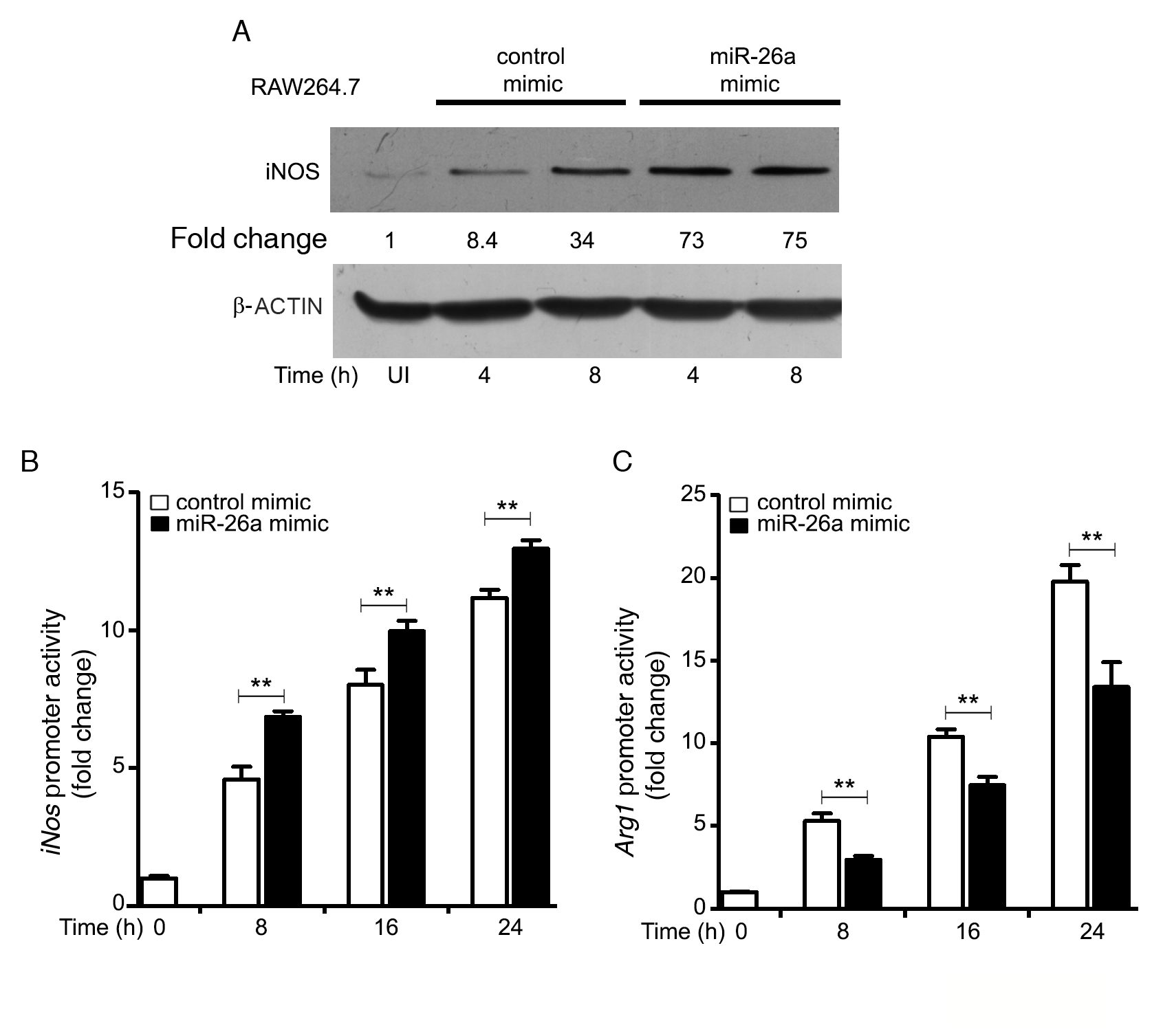

Supplement: S4 Fig — (A) RAW 264.7 cells were transfected with either control or miR-26a mimic, infected with Mtb for the indicated periods of time, lysates were prepared and iNOS expression was analyzed by Western blotting. The blot is representative of the results obtained in two independent experiments. Intensities of bands were measured by densitometric scanning. The fold change in iNOS was calculated with respect to uninfected cells. (B,C) RAW 264.7 cells were transfected with either control or miR26a mimic along with iNOS (B) or Arg1 (C) promoter luciferase construct, followed by infection with Mtb for different periods of time. Cells were lysed and luciferase activity was measured. Results represent means ± SEM. **p<0.01. (TIF) [file ppat.1006410.s004.tif]

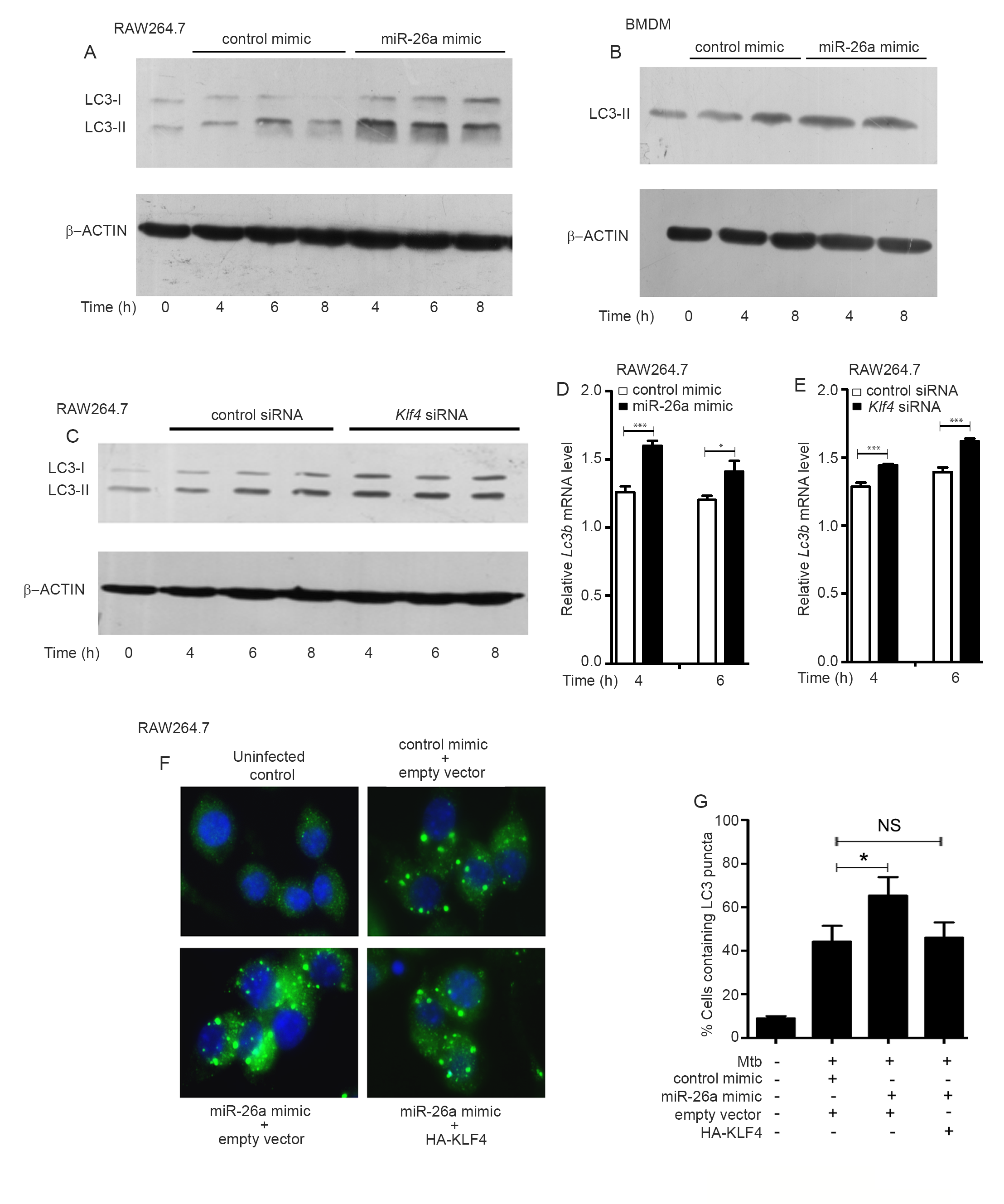

Supplement: S5 Fig — RAW264.7 (A,C,D,E) or BMDMs (B) were transfected with miR-26a mimic or control mimic (A,B, D) or with Klf4 siRNA (C, E); or with control mimic and empty vector or miR-26a mimic and empty vector or miR-26a +KLF4 expressing vector (F, G) prior to infection with Mtb. The conversion of LC3-I to LC3-II (A-C) or transcription of Map1lc3b (D,E) or the formation of LC3 puncta (F,G) was analyzed. Blots are representative of two separate experiments. For D, E and G, results represent means ± SEM, n = 3. * p< 0.05; ***p<0.001; NS: not significant. (TIF) [file ppat.1006410.s005.tif]

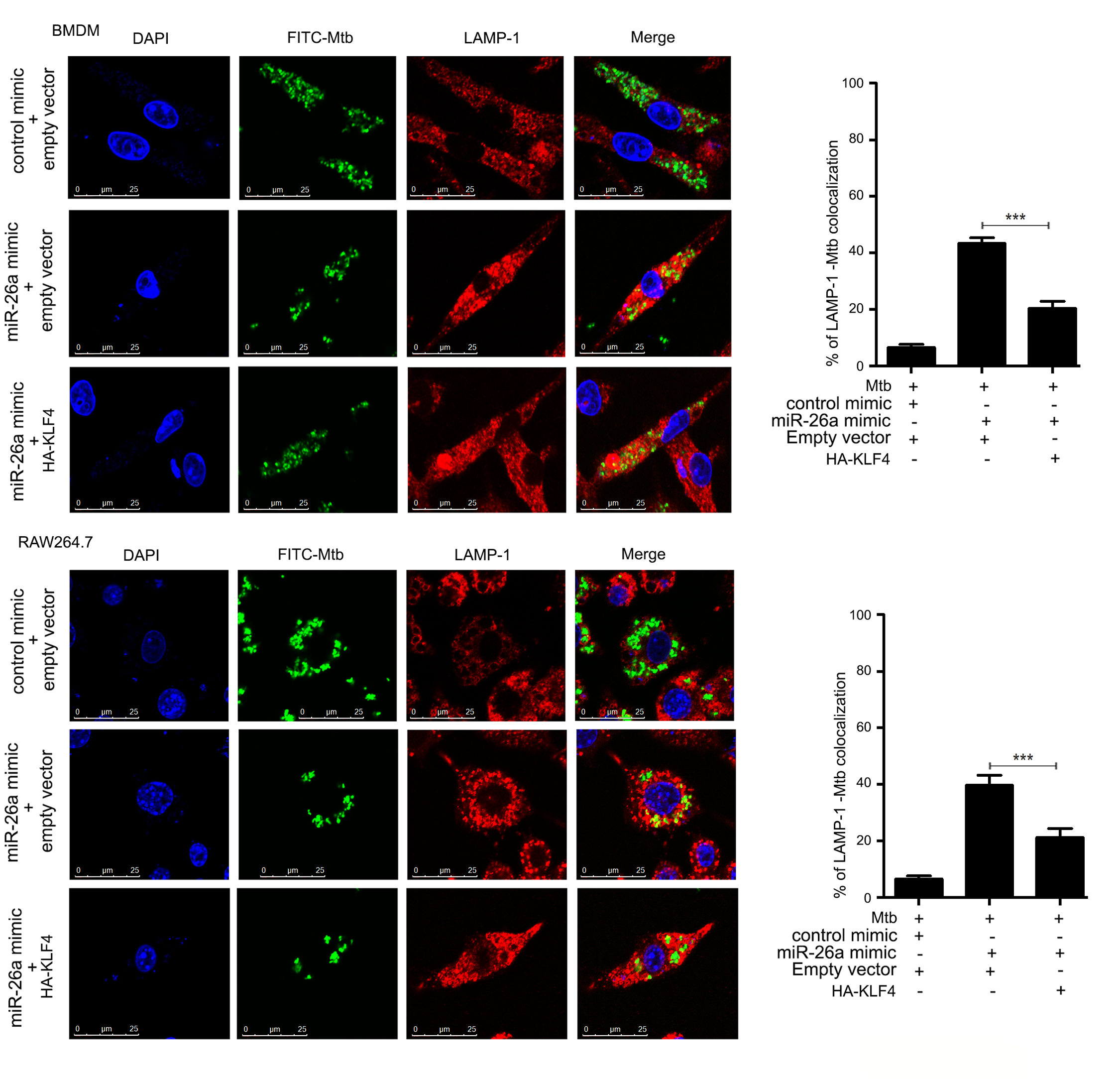

Supplement: S6 Fig — RAW264.7 cells or BMDMs were transfected with control mimic or miR-26a mimic for 24 h in combination with empty vector of KLF4 expressing plasmid as indicated. Cells were infected with FITC-labelled Mtb (green). After 24 h, cells were fixed and stained with LAMP1 antibody and Alexa-546 conjugated secondary antibody (red), and visualized by confocal microscopy. Nuclei were stained with DAPI. Colocalization of red and green fluorescence indicate that the mycobacteria reside in the lysosomal compartment. The panels on the right represent quantification of the results. The data represent three independent experiments in RAW264.7 and two independent experiments in BMDMs. *** p<0.001. (TIF) [file ppat.1006410.s006.tif]

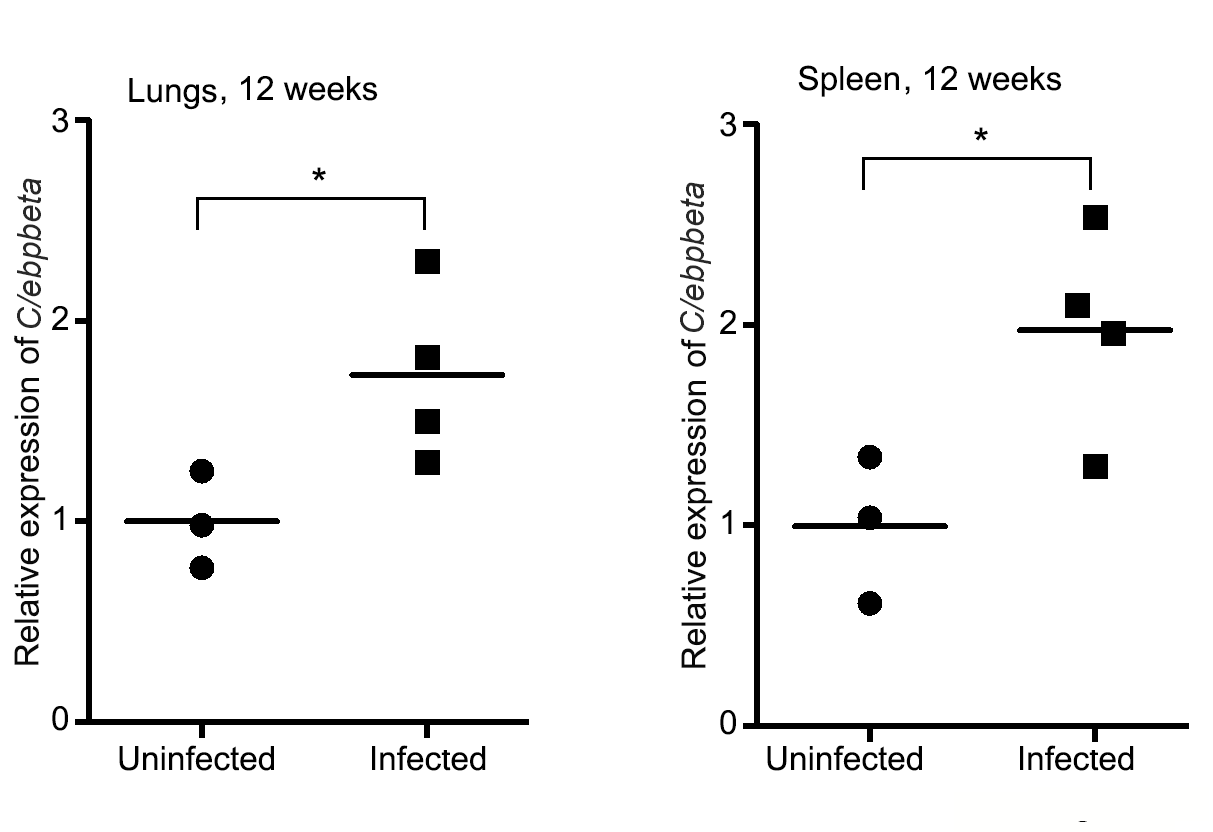

Supplement: S7 Fig — The relative expression of C/ebpbeta was quantitated by qRT- PCR. Each symbol represents one mouse. *p<0.05. (TIF) [file ppat.1006410.s007.tif]

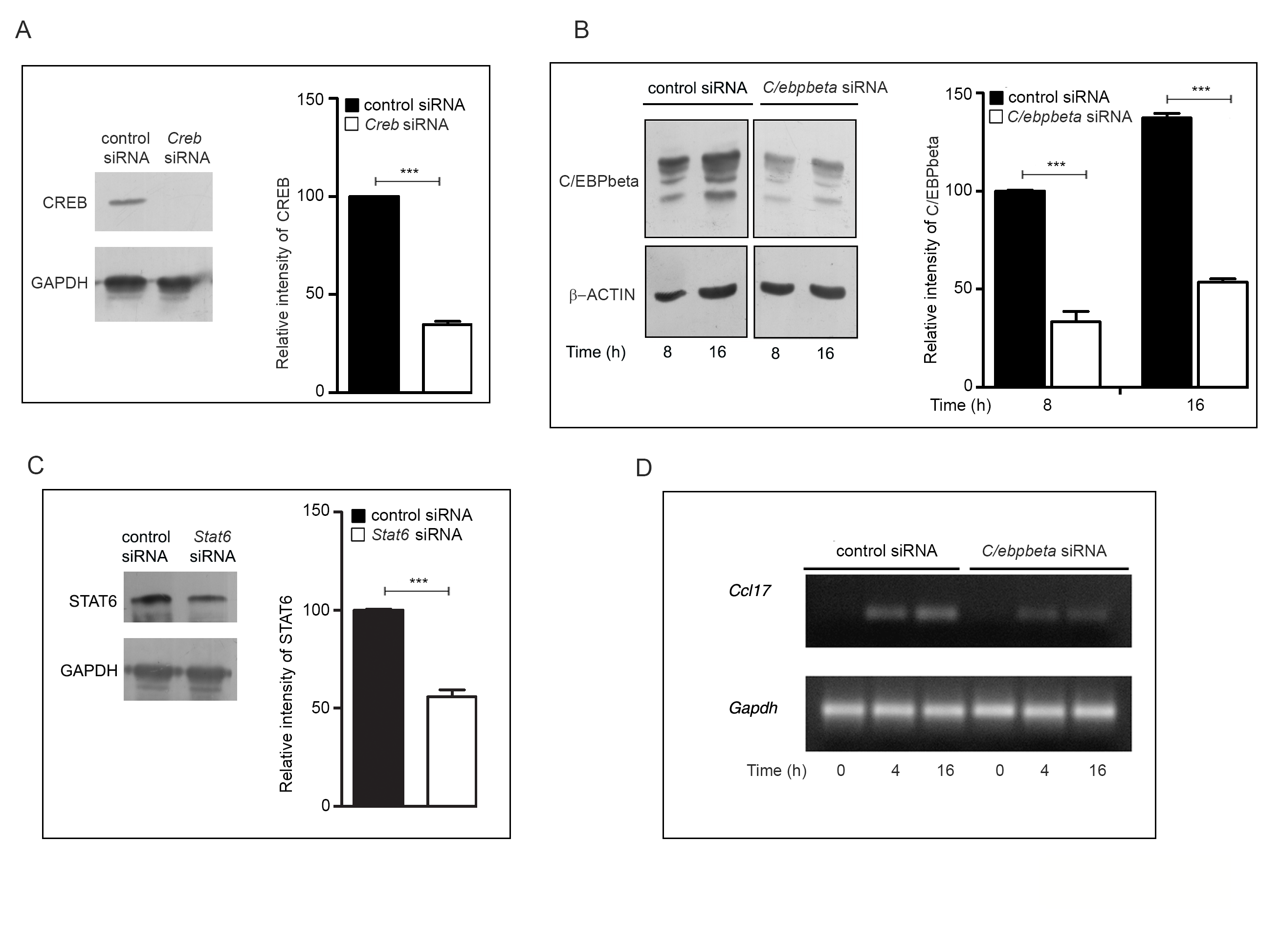

Supplement: S8 Fig — In the case of C/ebpbeta, transfected cells were infected with Mtb for the time periods indicated in panel B. Cells were lysed and immunoblotted with CREB (A), C/EBPβ (B) or STAT6 (C) antibody. Equal loading was confirmed by reprobing the blots with GAPDH β- actin antibody. Each blot is representative of the results obtained in three independent experiments. (D) RAW264.7 cells were transfected with either control or C/ebpbeta siRNA and infected with Mtb for different periods of time as indicated in the figure. RNA was isolated and the level of Ccl17 was quantitated by RT-PCR. For (A-C), intensities of bands were measured by densitometric scanning. Results are indicated in the bar plots. Means ± SEM. (n = 3). ***p< 0.001 (TIF) [file ppat.1006410.s008.tif]
