# Supplementary material for: MicroRNA 26a (miR-26a)/KLF4 and CREB-C/EBPβ regulate innate immune signaling, the polarization of macrophages and the trafficking of Mycobacterium tuberculosis to lysosomes during infection
Source: PLoS Pathog. 2017 May 30;13(5):e1006410. doi: 10.1371/journal.ppat.1006410 (PMC5466338; doi:10.1371/journal.ppat.1006410)
Supplement: S1 Table — (DOCX) [file ppat.1006410.s009.docx]

**S1 Table**. Fold change of a selected set of miRNAs that are downregulated during infection and are predicted to target KLF4

| miRNA | Fold Change (4h) | Fold Change (24h) |
| --- | --- | --- |
| mmu-miR-128-3p | 0.029 | 0.91 |
| mmu-miR-200b-3p | 0.97 | 0.26 |
| mmu-miR-200c-3p | 0.93 | 0.51 |
| mmu-miR-26a-5p | 0.67 | 0.39 |
| mmu-miR-26b-5p | 0.85 | 0.55 |
