# Supplementary material for: MicroRNA 26a (miR-26a)/KLF4 and CREB-C/EBPβ regulate innate immune signaling, the polarization of macrophages and the trafficking of Mycobacterium tuberculosis to lysosomes during infection
Source: PLoS Pathog. 2017 May 30;13(5):e1006410. doi: 10.1371/journal.ppat.1006410 (PMC5466338; doi:10.1371/journal.ppat.1006410)
Supplement: S2 Table — (DOCX) [file ppat.1006410.s010.docx]

| Gene Name | Primers: (F: forward, R: reverse)  5’--------------------------›3’ |
| --- | --- |
| Pri-miR-26a-1 | F: GGC CTC GTT CAA GTA ATC CA  R: GCC TTT AGC AGA AAG GAG GTT |
| pri-miR-26a-2 | F: TGC GGC TGG ATT CAA GTA AT  R: CTT CAT TGA GGG CAG ACC AT |
| Gapdh | F: GAA CGG GAA GCT TGT CAT CAA  R: CTA AGC AGT TGG TGG TGC AG |
| Klf4 | F: TGC CAG ACC AGA TGC AGT CAC  R: GTA GTG CCT GGT CAG TTC ATC |
| Cebpβ | F:ACCGGGTTTCGGGACTTGA  R:GTTGCGTCAGTCCCGTGTCCA |
| LC3b | F:CGTCCTGGACAAGACCAAGT  R:ACCATGTACTACAGGAAGCCG |
| Msr1 | F: CTG GAC AAA CTG GTC CAC CT  R: TCC CCT TCT CTC CCT TTT GT |
| Arg-1 | F: TTG GGT GGA TGC TCA CAC TG  R: TTG CCC ATG CAG ATT CCC |
| Socs3 | F: CCTTCAGCTCCAAAAGCGAG  R: GCTCTCCTGCAGCTTGCG |
| Irf4 | F: TCCGACAGTGGTTGATCGAC  R:CCTCACGATTGTAGTCCTGCTT |
| Ccl17 | F:AGTGCTGCCTGGATTACTTCAAAG  R:CTGGACAGTCAGAAACACGATGG |
| Ccl24 | F: TGTGACCATCCCCTCATCTTGC  R: AAACCTCGGTGCTATTGCCACG |

**S2 Table**

List of primers used for RT-PCR
