# Supplementary material for: MicroRNA 26a (miR-26a)/KLF4 and CREB-C/EBPβ regulate innate immune signaling, the polarization of macrophages and the trafficking of Mycobacterium tuberculosis to lysosomes during infection
Source: PLoS Pathog. 2017 May 30;13(5):e1006410. doi: 10.1371/journal.ppat.1006410 (PMC5466338; doi:10.1371/journal.ppat.1006410)
Supplement: S1 Text — (DOCX) [file ppat.1006410.s011.docx]

**S1 Text**

**Methods**

**Transient transfections**

For miRNA target validation, HEK293 cells were plated in DMEM without antibiotics in 24 well plates and grown for ~16 h before transfection. Cells were cotransfected with KLF4 3’UTR wild type (WT) or mutated (MUT) constructs and miR-26a mimic or control mimic using Lipofectamine 2000. RAW264.7 cells or BMDMs were plated in 35 mm plates or 12 well plates in antibiotic free medium one day prior to transfection. Cells were transfected with miR-26a mimic or control mimic (20 nM); or miR-26a inhibitor or control inhibitor (150 nM) using Lipofectamine 2000 following the manufacturer’s instructions. After 24 h of transfection, cells were infected with Mtb for the indicated time periods. Dhamafect 2 was used for transfection of siRNAs following the manufacturer’s instructions. After 48 h of transfection, cells were infected with Mtb. Plasmids were transfected using Lipofectamine 2000. Cotransfection of RAW264.7 cells with plasmids pCDNA3.1-HA-KLF4-FL or pGL3-mArg1 or pGL2-NOS2 or c-Myc-Ago2 along with miR-26a mimic or control mimic was performed using Lipofectamine 2000.

**Semi-quantitative and quantitative RT-PCR**

Total RNA was isolated using the mirVana miRNA isolation kit (Ambion). The RevertAid First Strand cDNA Synthesis Kit (Fermentas) was used for preparing cDNA. The expression levels of pri-miR-26a-1 and pri-miR-26a-2 were detected using semi-quantitative RT-PCR. PCR was carried out with the ExPrimeTaq DNA polymerase for 26 cycles. *Gapdh* was used as control. Quantitative PCR was carried out in triplicate using SYBR Green based real time PCR reactions in a 7500 Real-time PCR system (Applied Biosystems). A list of primers is given in Table S2. Expression of *Gapdh* was used for normalization. The relative expression was calculated using the comparative ΔΔCt method, and the values were expressed as 2^-ΔΔCt where ΔCt = CT_target_ – CT _Gapdh_ and Δ(ΔCt) = ΔCT _infected_ – ΔCt_uninfected_. The PCR primers of the indicated genes are listed in Table S2.

**Northern blotting**

For Northern blots, 5 µg total RNA was separated on 8 M Urea-15% polyacrylamide gels (Mini Protean II, Bio-Rad) at 200 V in Tris-boric acid-EDTA (TBE) Buffer. The gels were transferred to positively charged nylon membranes (Roche Applied Science) using a semi-dry transblot apparatus (Bio Rad) for 1 h at 200 mA. After transfer, RNA was crosslinked using EDC solution for 1 h at 60^0^ C.The membranes were pre-hybridized in UltraHyb hybridization buffer (Ambion) at 37^0^C for 1 h followed by hybridization with 0.5 nM DIG-labelled LNA-probe of miR-26a at 37^0^C for 16 h with slow rotation inside a hybridization oven. After hybridization, membranes were washed thrice with low stringent buffer (2X SSC, 0.1% w/v SDS) and twice with high stringent buffer (0.1X SSC, 0.1% w/v SDS) at 37 °C followed by blocking for 3 h at 25°C in blocking solution (Roche). After blocking, membranes were incubated with Anti-DIG-AP-Fab fragments (Roche) in blocking buffer at 25^0^C for 30 min. The membranes were washed with DIG-WASH buffer followed by incubation with CSPD (Roche) at 37^0^C for 10 min. Membranes were exposed to X-ray film and developed. Membranes were reprobed with DIG-labelled U6 probe as a loading control.

**Arginase activity assay**

Arginase activity assays were performed as described by Corraliza et al. [1]. Cells were lysed with 100 µl of 0.1% Triton X-100 by shaking for 30 to 60 min on a shaker at room temperature. Then 100 µl of 25 mM Tris-HCl (pH 7.5) was added. To 100 µl of this lysate, 50 µl of 10 mM MnCl_2_ was added and the enzyme was activated by heating for 10-15 min at 55^0^C. Arginine hydrolysis was analyzed by incubating the lysate with 100 µl of 0.5M L-arginine (pH 9.7) at 37^0^C for 15 to 120 min. The reaction was stopped by the addition of 600 µl of an acid mixture containing H_2_SO_4_ (96%) / H_3_PO_4_ (85%) / H_2_O at a ratio of 1:3:7. The concentration of urea was measured at 540 nm after addition of 40 µl 9% α-isonitrosopropiophenone (ISPF) and heating at 100^0^C for 45 to 60 min.

**Nitrite measurements**

After infection, supernatants were cleared by centrifugation before being assayed for NO by Griess reaction following the manufacturer’s instructions. Griess reagent was added to the supernatant and standards aliquoted in 96 well plates and incubated at room temperature in the dark for 10 min. Absorbance was read at 540 nm in an ELISA reader. Supernatant nitrite concentrations were calculated against a standard curve.

**Labelling of Mtb with fluorescein isothiocyanate (FITC)**

A suspension of Mtb was passed through a 26 gauge needle several times to break clumps, reconstituted in PBS containing 500 µg/ml FITC (Sigma) and kept at 4^0^ C under gentle rotation overnight. Thereafter FITC-labelled bacteria were washed three times in PBS and resuspended in DMEM for infections performed as described.

**Microarray data analysis**

Analysis of microarray data was done using GeneSpring GX 11.0. Raw signal intensities were normalized by percentile shift normalization to the 75^th^ percentile. ‘Absent’ flagged values were filtered out and probe sets with Present or Marginal intensity values were retained. The data were then subjected to ANOVA, with Benjamini–Hochberg FDR multiple testing correction, with a significance level of *p-*value < 0.05 and a fold-change value ≥ 2.0 to obtain the differentially expressed genes in Mtb infected macrophage cells at 4 hrs and 24 hrs in comparison to uninfected macrophages (UT).

From the gene expression dataset at different time points, TFs were screened using TRANSFAC [2] and QIAGEN’s Ingenuity Pathway Analysis (<https://www.qiagenbioinformatics.com/products/ingenuity-pathway-analysis/>). Morpheus [https://software.broadinstitute.org/morpheus/] was used to generate heatmaps for the TFs in untreated (UT) and Mtb infected macrophages.

**Analysis of differentially regulated miRNAs**

For compiling a list of miRNAs predicted to target KLF4, we used 2 different algorithms: PicTar (<http://pictar.mdc-berlin.de/>) [3] and TargetScan (<http://www.targetscan.org>) [4].

**References**

1. Corraliza IM, Campo ML, Soler G, Modolell M (1994) Determination of arginase activity in macrophages: a micromethod. J Immunol Methods 174: 232-235.

2. Matys V, Kel-Margoulis OV, Fricke E, Liebich I, Land S, Barre-Dirrie A, et al. (2006) TRANSFAC and its module TRANSCompel: transcriptional gene regulation in eukaryotes. Nucl. Acids Res. 34 (Database issue) D108-110.

3. Krek A, Grun D, Poy MN, Wolf R, Rosenberg L, Epstein EJ, et al. (2005) Combinatorial microRNA target predictions. Nat Genet 37: 495-500.

4. Agarwal V, Bell GW, NamJW, and Bartel D (2015).Predicting effective microRNA target sites in mammalian mRNAs. eLife. 4:e05005.
